# SWOOP: top-k similarity joins over set streams

**DOI:** 10.1007/s00778-024-00880-x

**Published:** 2024-12-23

**Authors:** Willi Mann, Nikolaus Augsten, Christian S. Jensen, Mateusz Pawlik

**Affiliations:** 1 Celonis SE, Munich, Germany; 2 University of Salzburg, Salzburg, Austria; 3 Aalborg University, Aalborg, Denmark

**Keywords:** Top-k similarity join, Streaming data, Set similarity

## Abstract

We provide efficient support for applications that aim to continuously find pairs of similar sets in rapid streams, such as Twitter streams that emit tweets as sets of words. Using a sliding window model, the top-*k* result changes as new sets enter the window or existing ones leave the window. Specifically, when a set arrives, it may form a new top-*k* result pair with any set already in the window. When a set leaves the window, all its pairings in the top-*k* result must be replaced with other pairs. It is therefore not sufficient to maintain the *k* most similar pairs since less similar pairs may become top-*k* pairs later. We propose SWOOP, a highly scalable stream join algorithm. Novel indexing techniques and sophisticated filters efficiently prune obsolete pairs as new sets enter the window. SWOOP incrementally maintains a provably minimal stock of similar pairs to update the top-*k* result at any time. Empirical studies confirm that SWOOP is able to support stream rates that are orders of magnitude faster than the rates supported by existing approaches.

## Introduction

The decreasing latency between the production of data, including humans and a broad range of sensors, and consumption of data renders streaming data increasingly prevalent. We consider streams where the elements of the streams are timestamped sets. Examples of such elements include social media texts (like tweets or news) that may be modeled as sets of words or *n*-grams [6, 13]; words in titles and bodies of email messages [20, 41]; retail point-of-sale transactions represented as sets of goods [55]; the clicks in user click-streams on a website [34, 39]; social media content represented by the sets of users that liked or consumed that content [27]; reviews represented as sets of terms for review spammer detection [12, 52]; or sets of events in event detection [15, 26, 52].

Such data streams may achieve very high frequencies. For example, Apple’s Siri user base may issue billions of requests per month; each request may be modeled as a set of words or other signatures. As another example, Twitter emits about half a billion tweets per day. To analyze such rapid data streams, new techniques must be developed that can keep up with high data emission rates, including peak rates. As new data items arrive in a stream, they are queued and processed in the FIFO order. When the processing cannot keep up with the stream rate, the queue grows and leads to waiting times for all subsequent data items. Delays between an event and its visibility in the result are critical in a situation when events require timely action, e.g., blocking a spamming email account [14]. A heuristic approach that dismisses data items to keep up with the stream rate leads to arbitrarily large errors: A single discarded data item may render incorrect all result pairs in the top-k list.

### Top-k Join over streams

We consider the problem of computing the top-*k* join in rapid data streams of timestamped sets with a sliding time window. A set falls into the window if the window covers its timestamp. We compute the *k* most similar pairs of sets in the window. As new data items arrive in the stream, the window moves, and the top-*k* result must be updated. The top-*k* join over streams may, for example, be used to recommend products based on recent point-of-sales transactions or click-stream data, to aggregate similar trending intelligent personal assistant requests to improve answer quality (e.g., by sharing successful interactions with users of similar requests), or to analyze information diffusion in streams of tweets [19].

Take, for example, the task of identifying similar trending topics on social media in New York City and London. For this purpose, we have access to streams of tweets from these cities. Each tweet can be represented as a set of words, after performing stemming and stop-word removal. The most recent tweet sets from the two streams are then continuously joined, and the *k* most similar set pairs are retained. This yields an overview of the most similar tweets from the two cities. Further processing may be applied to the top-*k* result, for example, tag clouds may be generated.

The top-*k* join with a sliding window is useful also for static data, where the window covers all data elements whose timestamp falls within the window. The window moves over the static data, and the join result is the union of top-k set pairs from every position of the window. For example, consider an ERP system in which users scan and upload documents and where near-duplicate documents should be detected (e.g., to avoid paying a bill twice). Each document is represented by a set of words resulting from an OCR process. Computing all pairs of near-duplicate documents in the entire database will typically lead to many irrelevant result pairs since documents of interest are uploaded within a small time frame. Therefore, only pairs within a given time window should be considered.

We model a *stream* as a sequence of $( set , timestamp )$ pairs with monotonically increasing timestamps. A time window *W* with duration *w* slides over the stream. Only pairs of sets with timestamps covered by the window are considered. As the window moves, newly covered sets become part of window *W* (or *enter* the window), and sets *expire* as they get too old and their timestamps are no longer within the window.

### Maintaining the join result

The top-*k* join result must be kept up-to-date when time passes and such changes occur. Maintaining the join result poses two main challenges. (1) *Candidate generation:* New sets that enter the sliding window may form a top-*k* pair with any of the sets currently in the window. (2) *Result expiration:* When sets expire, all their pairings become invalid. Expired pairs among the top-*k* must be removed, and replacements must be found to maintain a correct join result. We next discuss these challenges in detail.

#### Example 1

**Fig. 1:**
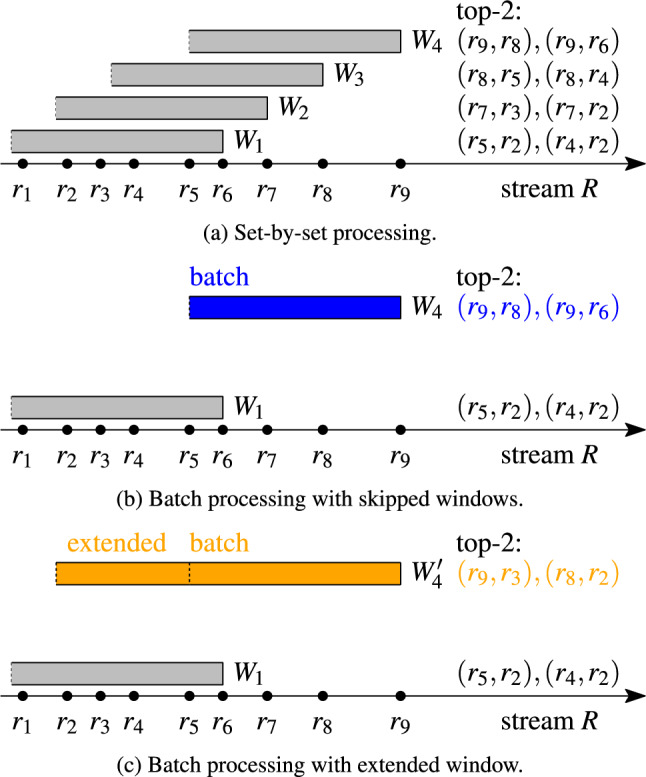
Batch processing leads to incorrect results

Fig. 1a shows how a top-2 result evolves as the sets covered by the window change. The horizontal axis represents a stream *R* of sets ($r_1$–$r_9$, $r_9$ is the youngest set). The bars symbolize the duration of a window sliding over the stream. All covered sets are included in the window. The set pairs to the right of each window are the top-2 result pairs in that window, e.g., $(r_7,r_3)$ and $(r_7,r_2)$ are the top-2 pairs in window $W_2$. Figure 1a shows that a single new set in the window can form pairs that are more similar than all pairs in the previous window: The new set $r_7$ in window $W_2$ forms two new pairs that are more similar than the top-2 pairs of window $W_1$. Moreover, set pairs become invalid as the window moves and sets expire: When the window moves from $W_2$ to $W_3$, sets $r_2$ and $r_3$ expire and the top-2 pairs of window $W_2$ are no longer valid in window $W_3$. Therefore, the entire top-*k* result may be invalidated by a window move.

**Candidate generation.** A new set that enters window *W* may form a pair with any of the |*W*| sets in *W*. In rapid streams, the sliding window may contain hundreds of thousands of sets, so computing the similarity between each new set and all sets in the window does not scale to fast stream rates. Well known similarity join techniques for static set collections rely on inverted list indices [3, 5, 23, 43, 46] that store a posting list of candidate sets for each set element called *token* (or for each signature [11]). Many techniques used in static scenarios, where all sets are known upfront, cannot be used for streams, e.g., we cannot order tokens by their frequency, nor can we process and index sets in non-decreasing size order. Further, an index for streams must remove expired sets, which is expensive in indexes for static data. Finally, core technologies like the prefix filter [7] that are leveraged in the static context use a threshold, whereas in our scenario a top-*k* result is required.

Algorithms for top-*k* joins over static collections of sets have been proposed [8, 40, 45, 53]. A fundamental assumption of these approaches, which is leveraged for both pruning and index construction, is that all sets are known upfront. There is no obvious way to adapt the static top-*k* join to our dynamic setting with frequent new and expiring sets. As we show in our empirical evaluation, reevaluating the static top-*k* join for each new set in the window does not scale. Note that a reevaluation after each new set is necessary. A heuristic approach that processes the new sets in batches by moving the window by a larger step to improve the performance may introduce a large error.

#### Example 2

Fig. 1 illustrates the problems of batch processing. Figure 1a shows that each time the window moves, the entire top-*k* result of the previous window may become invalid. In Fig. 1b, sets $r_7$, $r_8$, and $r_9$ are processed together in a batch, which leads to the final top-*k* pairs of $W_4$ ignoring the two intermediate results for $W_2$ and $W_3$. In order to cover the sets of the skipped windows, one could increase the window duration accordingly. This, however, may result in incorrect top-*k* pairs: In Fig. 1c, we extended window $W_4$ to cover all sets of the skipped windows ($r_2$ to $r_5$) and obtain top-*k* pairs that do not exist in any of the exact windows.

**Result expiration.** As time passes and the sliding window moves, sets leave the sliding window and expire. All pairs in the top-*k* result containing an expired set must be removed and replaced by other pairs. It is therefore insufficient to keep only the top-*k* pairs. Rather, a *stock* of less similar replacement pairs must be maintained. The total number of replacement pairs that can be formed is quadratic in the number of sets in the window. Hence, maintaining all such pairs is inefficient for large sliding windows or rapid streams. Only *relevant* pairs that are in the current top-*k* list or may be required later to maintain a correct top-*k* result should be stored.

**Fig. 2:**
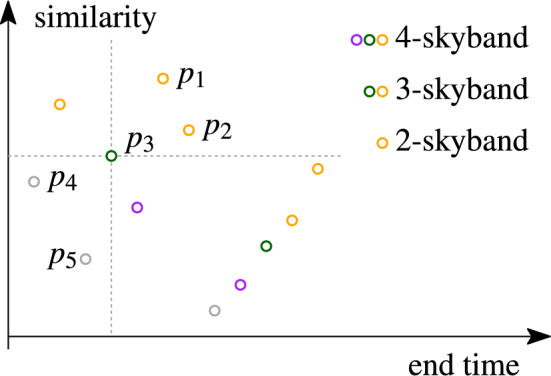
Example stock (not minimal) of 12 set pairs and *k*-skyband for different values of *k*

The stock that stores only relevant pairs is defined by the so-called *k*-skyband, which was introduced by Papadias et al. [31] and shown to be *minimal* for maintaining the top-*k* results [37]. The minimal stock consists of $O(k\cdot |W|)$ set pairs. To illustrate the skyband, we represent the set pairs as points in two-dimensional space. The first dimension is the *similarity* of the two sets in the pair; the second dimension is the *end time*, which is the earliest point in time when the pair becomes invalid, i.e., the older set in the pair falls out of the window. We say that one pair *dominates* another if it is both more similar and has a larger end time (with a tie allowed in one of the dimensions). Then, the *k*-skyband consists of all pairs that are dominated by at most $k-1$ other pairs.

#### Example 3

Fig. 2 shows an example stock of set pairs. This stock is not minimal for $k=2,3,4$. The color-coded circles represent set pairs in two dimensions: similarity (Y-axis) and end time (X-axis). The 2-skyband consists of the orange set pairs. The *k*-skyband is a subset of the $(k+1)$-skyband, e.g., the green pairs of the 3-skyband are not in the *k*-skyband for $k=2$ because each of them is dominated by more than $k-1$ pairs. The minimal stock for a specific value of *k* stores exactly the *k*-skyband pairs.

Consider pair $p_3$: Pairs $p_1$ and $p_2$ dominate $p_3$, $p_3$ dominates $p_4$ and $p_5$. All other pairs do neither dominate $p_3$, nor are dominated by it.

The state-of-the-art technique for computing the minimal stock, i.e., for identifying the relevant pairs among all pairs that can be formed in a given window *W*, is SCase [37]. Unfortunately, SCase does not support incremental updates. Therefore, the stock must be recomputed from scratch for each new set in the stream. A new set constitutes |*W*| new pairs which, together with the $O(k\cdot |W|)$ pairs stored in the stock, must be processed to recompute the stock. As our experimental evaluation confirms, SCase does not scale to rapid streams, and new approaches are required.

### Limitations of prior work and contributions

The focus of this paper is an exact and efficient solution for top-*k* similarity joins over streaming sets. Current solutions are either based on the repeated execution of static top-*k* joins or the stream join framework SCase [37]. We identify three key problems of the state of the art and highlight the contributions of our SWOOP algorithm.

*Static solutions are too slow (or incorrect).* Top-*k* join techniques for static scenarios cannot be easily extended to the stream setting as they assume all sets being known upfront. Recomputing the static top-*k* join for each new window does not scale to rapid streams as we show in our experimental evaluation. Batch processing of the sets leads to missing or even incorrect result pairs. Our SWOOP algorithm is exact and does not use batch processing to improve performance.

*Large number of candidates.* Each new set in the stream forms |*W*| new candidate pairs that may be relevant for the current or a future top-*k* result.^1^ SCase forms all these pairs, computes their similarities, and verifies their relevance: If a pair is dominated by at least *k* pairs in the stock, it will never be part of the top-*k* result and is therefore irrelevant. To identify the relevant pairs, SCase builds a so-called *k*-staircase: the set of points forming the *k*-skyband boundary. Only pairs above that boundary are relevant. SCase maintains the *k*-staircase in an additional data structure that is recomputed for each new set on the stream. Our SWOOP algorithm avoids touching each set pair and efficiently generates a small number of candidates using an index. We implement the *k*-staircase boundary as a lower bound function and integrate the lower bound and a new upper bound into the candidate index to further reduce the number of candidates. Only for the pairs resulting from the index lookup the similarity value is computed. The cost of updating our index in response to new or expiring sets is independent of the index size.

*Frequent recomputation of the stock.* The minimal stock of relevant pairs must be maintained efficiently. SCase recomputes the stock from scratch for each new set on the stream. We propose a novel technique to incrementally update the stock; this technique is not limited to streams of sets but is applicable to general stream join frameworks [37]. We show experimentally that the incremental stock update scales to stream rates that are up to ten times faster than the rates processed by SCase. When combined with the candidate index, we achieve speed-ups of up to three orders of magnitude compared to SCase.

In summary, we make the following key contributions:

- We present SWOOP, a novel algorithm for continuous top-*k* set similarity joins over streams. Two salient features of SWOOP are (1) the efficient generation of a small set of candidates when new sets enter the sliding window and (2) the incremental maintenance of a minimal stock to deal with new and expiring sets.
- We introduce the concept of *well-behaved similarity functions* to accurately characterize the applicability of SWOOP. All standard set similarity functions are well-behaved, including Overlap, Jaccard, Cosine, Dice, and Hamming.
- We present a solution to contend with the absence of so-called token frequency maps in streams; we particularly target difficult streams with very skewed token distributions.
- We report on an extensive empirical study showing that SWOOP is capable of running orders of magnitude faster than the state of the art. Our study offers insights into the efficiency of SWOOP compared to SCase [37], static top-*k* join [45], and a baseline solution.

**Outline.** Sect. 2 covers the related work and Sect. 3 defines the problem. Section 4 introduces the stream join framework and a baseline solution. Section 5 defines well-behaved similarity functions. Section 6 introduces the candidate generation algorithm, including the handling of difficult datasets. Section 7 covers the maintenance of the join result. Section 8 reports on our empirical study. We finally draw concluions in Sect. 9.

**Table 1:** Overview of related works

Input	Similarity query type			
	Top-k join	Top-k objects	kNN	Threshold based
Stream	SWOOP, [37]	[18, 28, 32, 51, 59]	[2, 4, 49]	[10, 30, 52]
Static	[8, 17, 33, 40, 45, 53, 54]	cf. Related Work in [28]	[21, 56, 60]	cf. [24] for a survey

## Related work

We categorize related works based on the following two aspects and provide an overview in Table 1.

- **Query type**. We address *top-k joins* and cannot leverage assumptions made by *top-k objects* (global score) or *threshold-based approaches* (user-defined similarity threshold).
- **Input data type**. We focus on *stream data*. Solutions for *static data* cannot be applied to streams other than executing a static algorithm for each new item on the stream.

### Top-k similarity joins

The *top-k similarity join* returns the *k* most similar pairs of items from a collection of items. The focus of this work is on top-*k* similarity joins over a *stream of sets* (shaded cell in Table 1).

*Stream data.* To the best of our knowledge, the only solution for top-*k* similarity joins in a streaming setting is SCase by Shen et al. [36, 37]. SCase is a generic framework for computing the top-*k* most similar pairs over sliding windows of object streams. For a given object type, a similarity function is supplied by the user.

The main differences between SCase and our SWOOP algorithm can be summarized as follows:

*(1) Candidate generation.* When a new set enters the window, SCase forms a candidate pair between the new set and each set in the window, i.e., the number of candidates grows linearly in the window size. For each candidate pair, the set similarity must be computed. SWOOP uses a novel index to retrieve only a small number of sets from the window for candidate generation, and the incremental index update is independent of the window size.

*(2) Stock maintenance.* SCase maintains a minimal stock by recomputing it from scratch for each new set on the stream. SWOOP uses a novel, merge-based technique to incrementally update the stock. Only the relevant set pairs are inserted, and the irrelevant pairs are removed in a local scan of the affected region in the stock.

We evaluate SCase experimentally: Due to the large number of candidates and the overhead for maintaining the stock, SCase cannot keep up with the high set rates of rapid streams. The set rate degrades almost linearly with the number of sets in the window, limiting the maximum window duration.

*Static data.* In a static setting all sets are known upfront. To apply a static algorithm to our problem, it must be reexecuted for each position of the the sliding window.

TopkJoin by Xiao et al. [45] extends the ideas of threshold-based similarity joins [3, 46] to the top-*k* setting. The threshold-based similarity join retrieves all pairs of sets that satisfy a user-defined similarity threshold. The high-level idea is to execute a threshold-based similarity join for increasing threshold values, avoiding, however, redundant computations. Yang et al. [53] investigate the benefit of probing multiple set elements in each step, while Wang et al. [40] focus on tuning individual steps of TopkJoin, e.g., the recursion depth of the suffix filter [46]. We experimentally compare our SWOOP algorithm to static approaches by reexecuting TopkJoin in response to each change of the sliding window.

A number of other static top-*k* join algorithms have been proposed that are, however, not applicable to the scenario discussed in this paper.

Ilyas et al. [17] compute top-*k* join queries in relational databases. Tuples are joined on equality, and a top-*k* ranking is computed for the joined tuples. The algorithm assumes a rank on the tuples of the input relations and a monotone combining ranking function that derives the rank of a joined tuple from the rank of the join partners. In our setting, there is no rank associated with the input tuples (sets) and there is no monotone combining ranking function: the rank of the joined tuples (pairs of sets) is computed from the similarity between the sets. As opposed to the static setting, our join result changes based on the content of the sliding window.

Qi et al. [33] compute a top-*k* similarity join on the *string* attributes of two relations. Two tuples match if the distance between the strings is within a user-specified threshold. The rank of the tuple pairs is based on a monotone combining function on numeric score attributes, which are assumed to be present in the input relations. In our scenario, the user does not specify a similarity threshold; the ranking is based on set similarity rather than a numeric score in the input tuples; and we consider sets rather than strings.

Cheema et al. [8, 9] propose a framework for top-*k* joins on multiple attribute values. The user specifies one global and multiple local scoring functions. The local scoring function ranks pairs of tuples on a specific attribute value; the global rank is computed from the local scores. An order is assumed on the ranked attributes, and the local scoring function must be *loosely monotonic* (a generalization of monotonicity) in that order. An example of a loosely monotonic function that is not monotonic is the absolute difference, $|a-b|$ between two numbers *a*, *b*. We cannot sort the sets in our scenario so that their similarity is loosely monotonic in the sort order.

Zhang et al. [54] propose a top-*k* join between two collections of sets, where the set elements are weighted multidimensional vectors. The similarity between the sets is the so-called $\phi$-quantile distance, which is computed in two steps. First, the vector pairs of two sets are ordered by their increasing Euclidean distance. Second, the vector pairs are scanned until the sum of the scanned weights exceeds $\phi$: the last scanned pair defines the $\phi$-quantile distance. In our scenario, the similarity is based on the overlap between sets.

Hu et al. [16] investigate the top-*k* spatio-textual similarity join that considers both the textual relevance and the spatial proximity of the tuples. Spatio-textual signatures are proposed: if two objects are among the top-*k* pairs, they must share at least one signature. Zhu et al. [57] compute the *k* closest pairs between two collections of static and moving spatial objects. Whenever the location of a moving object changes, the top-*k* result must be updated. Our work does not assume spatial attribute values.

### Top-*k* objects

The *top-k objects* query finds the *k* objects with the highest scores. A fixed score is assigned to each object. In our scenario, the individual objects (sets on the stream) do not have a score, instead, the score is computed for pairs of objects.

*Stream data.* A number of studies compute top-*k* queries over streams of objects with a fixed score. All objects have the same lifetime, which is determined by a sliding window. In our setting, the lifetime of a set pair is determined by the lifetimes of both sets and varies between the pairs, which poses additional challenges.

Mouratidis et al. [28] compute top-k objects in a stream of sets using a grid structure to index valid objects. They identify a subset of grid cells in which an update invalidates the current top-k result. A stock of objects for later updates is maintained. Compared to our scenario, older objects cannot dominate younger ones, which simplifies the task. Yang et al. [51] improve over [28] by identifying the minimal set of objects in the window that is both necessary and sufficient to answer top-k queries as the window slides. This significantly reduces the number of object that must be kept in memory and must be considered to update the top-*k* result. Zhu et al. [59] extend [28] by partitioning the window into sub-windows. In each subwindow, only the candidates with the highest scores are maintained. The partitioning dynamically adjusts to the data in the stream to reduce the number of candidate updates as the window slides. The partitioning is improved by Jiang et al. [18]: After testing the predictability of the streaming data, they construct a suitable model to predict the distribution of the object scores.

Zou et al. [58] and Pripužić et al. [32] propose approximate solutions for the top-*k* objects query over streams, Wahab et al. [38] deal with uncertain streams. We focus on exact solutions of the top-*k* *join* problem over streams of sets.

*Static data.* For solutions to top-*k* object queries on static collection we refer to the discussion in [28].

### *k*-nearest neighbors

The *k*-nearest neighbors query (*k*NN) identifies the *k* most similar objects for a given query object.

*Stream data.* Böhm et al. [4] address the problem of multiple *k*NN queries: The user specifies a set of query objects for which the *k* most similar objects from a stream must be maintained. Stream updates that cause a change in the result are reported. Xu at al. [49] and Yamazaki and Koga [50] treat the *n* most recent items on a stream of set elements as an evolving set query. Whenever a new item is added to the stream, the set query changes and the top-*k* result over a static collection of sets is updated. Amagata et al. [2] compute a set *k*NN self join in a dynamic environment, where set elements change over time. The set *k*NN self join returns the *k* most similar sets for each set in the collection.

*Static data.* A number of works deals with *k*NN queries over static collections of sets [21, 56], whereas we study top-*k* joins over streams. The focus of Zois et al. [60] is the performance of *k*NN queries in multi-core architectures.

### Threshold-based approaches

*Threshold-based approaches* return pairs of items that are within a user-specified similarity threshold. The threshold can be used to prune irrelevant candidate pairs. In our top-*k* scenario, we cannot leverage a user-defined threshold.

*Stream data.* Yang et al. [52] study the threshold-based similarity join on a stream of sets in a distributed setting. They focus on distributed computation paradigms to reduce data replication, communication cost, and improve load balancing. The join is computed between each incoming set and all sets in the stream history, whereas we assume a sliding window model and compute the self-join in each window.

Morales et al. [10] join streams of vectors and return all pairs of vectors that are within a user-defined similarity threshold. The similarity is assessed by extending the Cosine similarity to also consider the age of pairs using a pre-defined time-decay parameter. Pacífico and Ribeiro [29, 30] use the time-decay model to join streams of timestamped sets. These algorithm cannot be applied in our setting because the time-decay cannot be modified to simulate a sliding window, and we cannot leverage a fixed threshold.

*Static data.* Several works deal with threshold-based set similarity joins on static data [11, 24, 43]. Deng et al. [11] leverage the pigeonhole principle on set partitions to prune candidates. A particularly successful concept is the so-called prefix filter [7], which has been exploited in many set join algorithms [3, 5, 23, 35, 41, 43, 47]. Neither set partitioning nor prefix filtering can be applied in our top-*k* settings as they require a fixed threshold. Wang et al. [42] study a threshold-based similarity join on two windows that slide over a query and a document, respectively; the window defines a set of fixed length. In our setting, the sliding window covers all valid sets in the stream at a specific point in time, and the lengths of the sets in the stream may vary.

## Problem setting and definition

**Basic concepts.** A *stream* *R* is a sequence of tuples $$(r_i, t_i)$$, where $$r_i$$ is a set and $$t_i$$ is a timestamp. The timestamp monotonically increases with the sequence number, i.e., for any two tuples $$(r_i,t_i)$$ and $$(r_j,t_j)$$, $$i>j\Rightarrow t_i\ge t_j$$. A *sliding window* *W* over stream *R* contains all tuples of *R* that at time $$t_J$$ are no older than *w*: $$W(R,t_J)=\{(r_i,t_i)\in R \mid t_J- w < t_i \le t_J\}$$; *w* is the window duration; the *join time* $$t_J$$ is the position of the sliding window on the stream and defines the largest point in time covered by the window. The sets in the sliding window are called $$valid $$. Two valid sets form a *valid pair*. A pair $$(r_i,r_j)$$ is valid for as long as both $$r_i$$ and $$r_j$$ are covered by the window. Table 2 summarizes the notation.

**Table 2:** Notation overview

*R*	Stream of sets
$r_i$	*i*-th set in stream *R*
$t_i$	Timestamp of set $r_i$
*W*	Sliding window on *R*
*w*	Window duration
$t_J$	Join time
*T*	Top-*k* list
$p=(r_i,r_j)$	Pair of sets $r_i$ and $r_j$
$\textit{sim}(p)$	Similarity of sets in *p*
$\textit{end}(p)$	End time of pair *p*
$\uptau$	Set similarity threshold
|*r*|	Cardinality of set *r*

**Window join.** To simplify the presentation, we discuss a self join scenario where a stream is joined to itself; with minor modifications, all the techniques presented in this paper extend to the general case of joining two different streams.

The top-*k* set similarity join in sliding window *W* returns the *k* most similar pairs of sets from stream *R* that are valid at the time the query is issued. Various functions have been proposed to assess the similarity between sets, e.g., Jaccard, Cosine, or Dice [45]. In Sect. 5 we define the concept of *well-behaved* set similarity functions that characterize the functions supported by our solution.

### Definition 1

*(Top*-*k* *Set Similarity Join)*

Given a sliding window *W* over stream *R* and a set similarity function $\textit{set\_sim}(\cdot ,\cdot )$, the top-*k* set similarity join returns a set of *k* valid set pairs $T = \{p_1, p_2, \ldots , p_k \}$ from $W \times W$, such that for all $(r_i,r_j)\in T$:

1. $$i>j$$,
2. $$r_i \cap r_j \ne \emptyset $$,
3. For all valid pairs $(s_i,s_j)$ not in *T*:$\textit{set\_sim}(s_i,s_j)\le min_{(r_i,r_j)\in T} \{\textit{set\_sim}(r_i,r_j)\}$.

$|T|<k$ if fewer than *k* pairs qualify.

Condition (1) eliminates symmetric pairs such that only one of $(r_i,r_j)$ and $(r_j,r_i)$ is included in *T*. Condition (2) discards pairs that have no element in common. Condition (3) ensures that the pairs in *T* are the most similar pairs in the sliding window.

The join in Definition 1 is a one-time query because it is executed once. We consider the *continuous* variant of the query that maintains an up-to-date result from when it is started until when it is stopped. As time passes, the window *W* slides over the stream. Some sets leave *W* (expire), and new sets enter *W*. The join result *T* must be kept up-to-date when such events occur. A set $$r_i$$ that enters window *W* at time $$t_i$$ forms a new valid pair with all other sets $$r_j$$ in *W*, where $$i>j$$. A new pair enters the join result if it is sufficiently similar. When a set $$r_i$$ expires, all pairs that contain $$r_i$$ become invalid. Invalid pairs must be removed from *T*, and they must be replaced by valid pairs. A pair $$(r_i,r_j)$$ is valid from time $$t_i$$ (when the younger set enters the window) until its *end time* $$t_j+w$$ (when the older set leaves the window), i.e., its validity interval is $$[t_i, t_j+w)$$. The validity interval of a pair always contains the join time $$t_J$$.

### Example 4

**Fig. 3:**
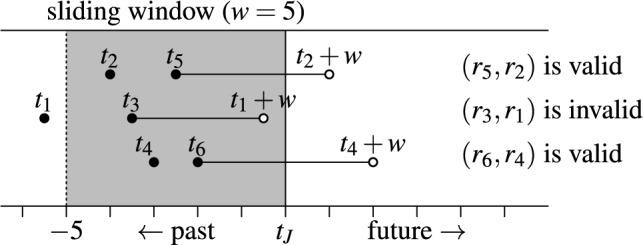
Valid and invalid pairs with their validity intervals

Figure 3 shows three set pairs $(r_5,r_2)$, $(r_3,r_1)$, $(r_6,r_4)$. The sliding window *W* (shaded in gray) has duration $w=5$. The join time $t_J$ positions the window on the timeline. $t_i$ is the timestamp of set $r_i$. The validity intervals of pairs are marked with horizontal lines, e.g., pair $(r_5,r_2)$ is valid from time $t_5$ until its end time $t_2+w$. Pair $(r_3,r_1)$ is invalid because $r_1$ has already expired (is outside of the window) and the end time of the pair is smaller than the join time $t_J$. The top-*k* join query at time $t_J$ considers only the valid pairs $(r_5,r_2)$ and $(r_6,r_4)$.

**Problem statement.** Our goal is to solve the *continuous top-k* *set similarity join* over rapid streams using a sliding window. In particular, we aim at a solution that scales to high set rates even for large windows.

## Join framework and baseline

We introduce Algorithm 1, our framework for the continuous top-*k* join query on stream *R*, and cover its baseline implementation. The framework comprises two data structures, *window W* and *stock* $S$, and four operations.

**Algorithm 1:**
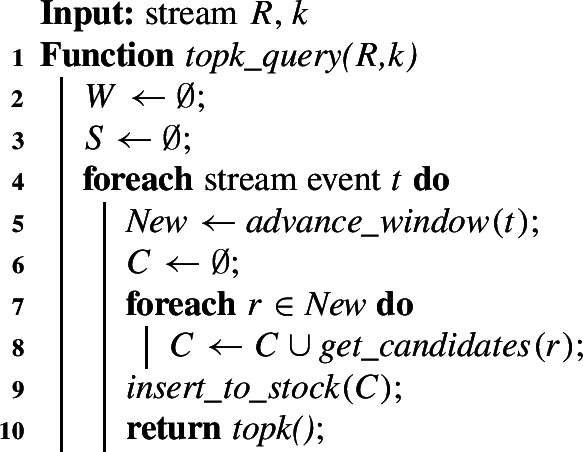
Framework of the top-*k* query.

*Window* *W* stores all sets of stream *R* covered by the sliding window at time $t_J$. *W* is used when evaluating the similarity between valid pairs of sets and when expiring sets as the join time $t_J$ increases (i.e., the sliding window is advanced). *W* is implemented as a FIFO queue that supports iterations and the usual peek/pop/push operations.

*Stock *$S$ maintains the join result *T* at time $t_J$. Additionally, it stores at least all *relevant pairs*. A pair is relevant iff it is in the minimal stock at some point in time. The minimal stock stores the minimum set of pairs that must be kept to serve as possible replacements in the top-*k* result when sets expire (cf. Sect. 1). $S$ stores four-tuples $(r_i,r_j,\textit{set\_sim}(r_i,r_j),t_j+w)$, where $t_j+w$ is the end time of pair $(r_i,r_j)$, i.e., the time when $(r_i,r_j)$ becomes invalid. $S$ is implemented as a binary search tree ordered by descending similarity of the set pairs, i.e., the top-*k* pairs are ranked first.

$\textit{advance\_window}(t)$ (line 5) moves the sliding window to the next *stream event* $t>t_J$ and advances the join time $t_J$ to *t*. A stream event is each point in time when an old set expires or a new set appears on the stream. The function removes all expired sets (if any) from window *W*, removes invalid pairs from stock *S*, and fetches all (if any) new sets $\textit{New}$ valid at time *t* from the stream and adds them to the window.

$\textit{get\_candidates}(r)$ (line 8) computes the similarity of each new set pair $(r,r')\in \textit{New}\times \{r'\mid r'\ne r \wedge r' \in W\}$. All pairs with the similarity greater than zero, $\textit{set\_sim}(r,r_j)>0$, are returned as the set of *candidates* *C*, which contains at least all new relevant pairs.

$\textit{insert\_to\_stock}(C)$ (line 9) inserts all candidate pairs *C* into the stock $S$ and ensures that the stock stores all relevant pairs. After the insertion, the stock $S$ contains the join result as of time $t_J$.

$\textit{topk}()$ retrieves the join result *T* at time $t_J$ by traversing the first *k* pairs in stock $S$ (or $|S|$ pairs if $|S|<k$). No stock update is required.

Algorithm 1 iterates over the stream events (line 4). At a stream event: (1) each expired set is removed from the window, and all invalid pairs are removed from the stock (line 5); (2) every new set in $\textit{New}$ is added to the window *W* (line 5); (3) the candidate pairs *C*, including at least all new relevant pairs, are computed (line 8) for every $\textit{New}$ set; (4) all candidates are inserted into the stock (line 9). Finally, $\textit{topk}()$ (line 10) reports the join result *T* at time $t_J$.

### Example 5

**Fig. 4:**
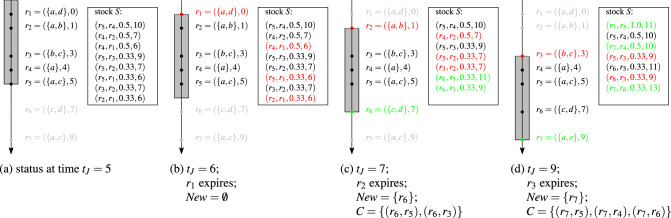
Processing sets by the baseline solution in our stream join framework. Window duration $w=6$

Fig. 4 shows three iterations (b)–(d) of the baseline implementation of our stream join framework. Part (a) shows the initial status at time $t_J=5$. The stream is represented by the vertical axis. The labeled dots on the stream axis are sets, the window is represented as a shaded rectangle. Sets covered by the window are valid. The stock elements are sorted by set similarity (Jaccard similarity in this example). Expired sets and invalid pairs in stock are marked in red. The expiring sets are at the top of the window in Figures (b)–(d). New sets and new entries in stock *S* are marked in green. In Figure (b), $\textit{advance\_window}(6)$ moves the window to the next stream event $t=6$ when set $r_1$ expires and thus invalidates three pairs in the stock: $(r_4,r_1)$, $(r_5,r_1)$, and $(r_2,r_1)$. In Figure (c), we advance to the next stream event $t=7$ when set $r_2$ expires and a new set $r_6$ appears on the stream. $\textit{get\_candidates}(r_6)$ computes two candidate pairs, $(r_6,r_5)$ and $(r_6,r_3)$, which are then ranked in the stock ($\textit{insert\_to\_stock}(\{(r_6,r_5),(r_6,r_3)\})$). The next stream event, $t=9$, is visualized in Figure (d).

*Complexity of baseline.* Stock $S$ is of size $O(|W|^2)$ and dominates the memory complexity. $\textit{insert\_to\_stock}$ runs in $O(|W|\log |W|)$-time since a new set must be paired with every set in *W*, and the new pairs must be inserted into the binary search tree $S$. $\textit{advance\_window}$ collects invalid pairs by scanning stock *S* in $O(|W|^2)$ time; removing an entry from stock has cost $O(\log |S|)=O(\log |W|)$. Finally, $\textit{topk}$ runs in optimal *O*(*k*) time.

*Outline of the SWOOP algorithm.* The inefficiency of the baseline solution arises from the many candidate pairs generated for each incoming set and the quadratic size of the stock, which must be maintained under frequent changes. We address these issues in the following sections. Section 5 characterizes the scope of our solution. Section 6 introduces a novel indexing technique to generate candidates, the *candidate index*, which needs to consider only a small fraction of the sets in window *W*. Section 7 proposes an efficient stock implementation that stores only $O(k\cdot |W|)$ pairs, is maintained incrementally, and interacts with the candidate index to limit the number of candidates.

## Supported similarity functions

Our solution works with the most common similarity functions, including Jaccard, Cosine, Dice, Overlap, and the Hamming distance, but is not limited to them. We introduce the concept of a *well-behaved set similarity function* to abstract from individual measures and identify the essential properties that a similarity function must satisfy to be applicable to our framework. In our correctness proofs, well-behavedness is the only assumption on the underlying set similarity function.

### Definition 2

*(Well-behaved set similarity function)*

A similarity function between two sets *r* and *s*, $\textit{set\_sim}(r,s)$, is *well-behaved* iff there is a function $\textit{sim}(x, y, o)$ such that $\textit{set\_sim}(r,s)=\textit{sim}(|r|,|s|,|r\cap s|)$ for overlap $|r\cap s|$ and set lengths |*r*|, |*s*|, and $\textit{sim}(x,y,o)$ satisfies the following properties:

1. $$\textit{sim}(x,y,0)=0$$;
2. $$\textit{sim}(x,y,o)=\textit{sim}(y,x,o)$$ (symmetric);
3. for all $o\le o'$, $\textit{sim}(x,y,o)\le \textit{sim}(x,y,o')$ (monotone in set overlap);
4. for all $y\le y'$, $\textit{sim}(x,y,o)\ge \textit{sim}(x,y',o)$ (antitone in set length);
5. for all $o\le o'$, $\textit{sim}(x,o,o)\le \textit{sim}(x,o',o')$ (monotone in subset length);

**Fig. 5:**
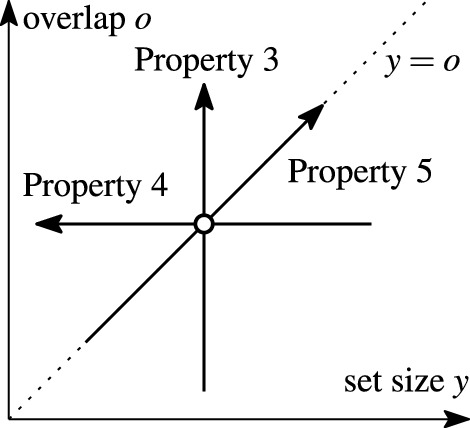
Monotonicity axes of function $\textit{sim}(x,y,o)$ (Properties 3–5 of well-behaved similarity functions)

### Example 6

Figure 5 illustrates the monotonicity properties of the function $\textit{sim}(x,y,o)$. Assuming a fixed set size *x*, the function is monotone in the overlap *o* (Property 3) as well as the subset length $y=o$ (Property 5) and antitone in the set length *y* (Property 4). For example, consider set $r=\{a,b,c\}$ of length $x=3$ and Jaccard similarity $\textit{sim}(|r|,|s|,|r\cap s|)=|r\cap s|/(|r|+|s|-|r\cap s|)$ (cf. Table 3).

*Property* 3: Let $y=|s|=2$. If $s=\{a,d\}$, the overlap $o=|r\cap s|=1$, and $\textit{sim}(x,y,o)=1/4$. Increasing the overlap to $o'=2$ must not decrease the similarity value: Let $s'=\{a,b\}$, $y'=|s'|=y$, such that $o'=|r\cap s|=2$; then, $\textit{sim}(x,y,o')=2/3$.

*Property* 4: Let $s=\{a,b\}$, $y=|s|=2$, $s'=\{a,b,d,e,f\}$, $y'=|s'|=5$. The similarity value must not increase with increasing set size: For both *s* and $s'$, the overlap $o=2$, and $\textit{sim}(x,y,o)=2/3>\textit{sim}(x,y',o)=2/5$.

*Property* 5: This property requires $y=o$, which entails $s\subseteq r$. Let $s=\{a\}$, $y=|s|=1$, $o=|r\cap s|=1$, $s'=\{a,b\}$, $y'=|s'|=2$, $o'=|r\cap s'|=2$. The increase of both set length and overlap must not result in a lower similarity value: In our example, $\textit{sim}(x,y,o)=1/3<\textit{sim}(x,y',o')=2/3$.

In Definition 2, each property of $\textit{sim}(x,y,o)$ can be translated into an equivalent property of the well-behaved similarity function $\textit{set\_sim}(r,s)=\textit{sim}(|r|,|s|,|r\cap s|)$. For example, Property 5 covers the case when one of the input sets is contained in the other, i.e., the overlap matches the size of the contained set: if $|s|=|r\cap s|\le |s'|=|r'\cap s'|$ (i.e., $s\subseteq r$, $s' \subseteq r'$) and $|r|=|r'|$, then $\textit{set\_sim}(r,s)\le \textit{set\_sim}(r',s')$.

Well-behavedness can analogously be defined for set distance functions. A set distance function, $\textit{set\_dist}(r,s)$, is well-behaved if there is a function, $\textit{dist}(x,y,o)$, such that $\textit{set\_dist}(r,s)=\textit{dist}(|r|,|s|,|r\cap s|)$, and the properties in Definition 2 hold for a complementary function $\textit{sim(x,y,o)}=\textit{dist}(x,y,0)-\textit{dist}(x,y,o)$.

### Lemma 1

The similarity functions Jaccard, Cosine, Dice, and Overlap, and the Hamming distance are well-behaved.

### Proof

Table 3 defines function $\textit{sim}(x,y,o)$ required by Definition 2 for each of the similarity functions. For the Hamming distance, Table 3 defines $\textit{dist}(x,y,o)$; due to $\textit{dist}(x,y,0)=x+y$, the complementary function of the distance $\textit{dist}(x,y,o)$ is $\textit{sim(x,y,o)}=2o$. $\square$

**Table 3:**
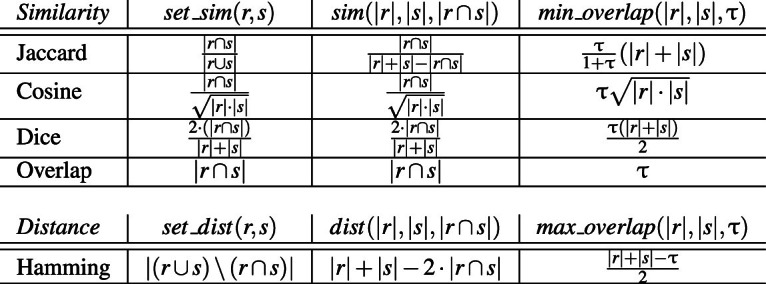
Examples of well-behaved similarity and distance functions

For well-behaved set similarity functions we can find a *minimum required overlap* such that $\textit{set\_sim}(r,s)\ge \uptau$ for a given similarity threshold $\tau$. Table 3 shows function $\textit{min\_overlap}(x,y,\uptau )$ that computes the minimum required overlap for various similarity functions and, analogously, for the Hamming distance.

### Lemma 2

Let $\textit{set\_sim}(r,s)$ be a well-behaved set similarity function between two sets *r* and *s*. There is a function $\textit{min\_overlap}(x,y,\uptau )$ that maps the lengths $x=|r|$, $y=|s|$ of any pair of sets *r*, *s*, and a set similarity threshold $\uptau \ge 0$ to the smallest overlap $o=|r\cap s|$ such that $\textit{set\_sim}(r,s)\ge \uptau$.

### Proof

For $\tau =0$, overlap $o=|r \cap s|=0$ according to Property 1 in Definition 2. For any $\tau >0$ we can find an minimum overlap that satisfies $\textit{set\_sim}(r,s)\ge \uptau$ as well-behaved similarity functions monotonically increase with the overlap for given set lengths $x=|r|$ and $y=|s|$ (Property 3 in Definition 2). $\square$

## Efficient candidate generation

When a new set enters the sliding window, it may cause the top-*k* result to change (cf. $\textit{advance\_window}(t)$, Sect. 4). Recall that such a new set triggers two operations in our join framework: $\textit{get\_candidates}(t)$ identifies all candidate set pairs *C* that may change the topk-*k* result at the current join time or in the future, $\textit{insert\_to\_stock}(C)$ inserts those candidates into the stock. This section focuses on efficient candidate generation.

**Limitations of the baseline.** The baseline implementation of our join framework generates candidates by pairing each new set $r_i$ with every set $r_j$ in the window (cf. Sect. 4). For each such pair the similarity value is computed, and all pairs with $\textit{set\_sim}(r_i,r_j)>0$ are candidates that are inserted into the stock.

Unfortunately, the baseline does not scale to large windows or rapid streams for two reasons: (1) The large number of similarity computations that are required; the cost of evaluating similarity functions, like Jaccard or Cosine, is linear in the set size. (2) The large number of candidate pairs that must be inserted into the stock, including many candidates that are irrelevant and will never contribute to the top-*k* result. The objective is to keep the candidate set small without missing any relevant pairs.

Note that the so-called dominance check introduced in the SCase algorithm [37] only addresses the number of stock insertions, but does not avoid iterating over all set pairs and computing their similarities, which we identify as a serious bottleneck (cf. Sect. 8).

### The candidate index

We introduce the *candidate index* *I*, an inverted list index that efficiently retrieves a small set of candidates. The index key is a set element, called *token*, that maps to a list of pairs $(r_j, t)$, where $r_j$ is a set that contains the key token and $t=t_j+w$ is the end time of $r_j$. We implement the index with doubly-linked lists. The sets in the lists are ordered by increasing end time, and only *valid* sets are stored in the index. When a new set $r_i$ enters the sliding window, the lists of all tokens in $r_i$ are accessed to retrieve candidates, and the index *I* is updated.

#### Example 7

**Fig. 6:**
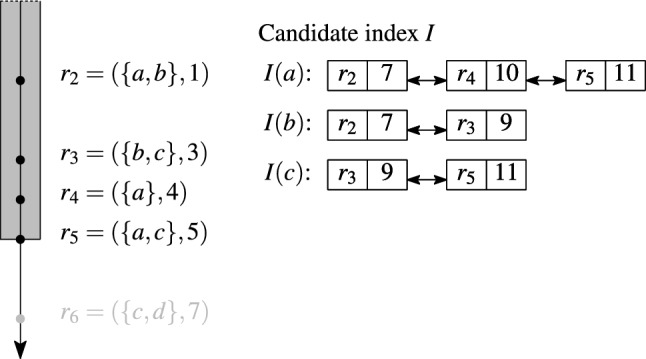
Candidate index. Window duration $w=6$

Figure 6 shows our candidate index for the valid sets $r_2$–$r_5$ at join time $t_J=5$ for a window of duration $w=6$. The numbers in the index are the end times of the sets, i.e., $t_j+w$, $2\le j\le 5$. The index stores three posting lists, one for each unique token in the valid sets.

Inverted indexes have also been used in static settings to reduce the number of candidates in set similarity joins. However, the techniques used in static scenarios are not applicable in the stream context. In static scenarios, all sets are known upfront and are preprocessed to support efficient indexing and effective candidate filters. For example, the tokens within a set are sorted by increasing frequency (to favor the prefix filter [7]), the sets are processed and indexed in non-decreasing length order (to support the length filter [3, 23]), and sets need not be removed as the index size is bound by the data size. In our streaming scenario, we cannot preprocess the data, and our index must support efficient updates as new sets arrive and old sets expire. New techniques are required to efficiently index streaming set data.

**Index lookup.** When a new set $r_i$ enters the window, we look up the candidate index *I*. For the lookup, we define a strict total order on the tokens and denote the $\rho$-th token of $r_i$ with $r_i[\rho ]$. The order is defined by the lookup order of the tokens in the index and can be different for each set.

We first discuss a naive use of our index: For each token $v\in r_i$, we retrieve the index list *I*(*v*). Each list is traversed from tail to head. Every *unseen* set in a list, i.e., a set which has not been encountered before during the lookup, forms a new candidate pair with $r_i$. Formally, a set $r_j\in I(r_i[\rho ])$ is *unseen* if:

1. $\rho =1$: $r_j$ appears in the index list of the first token of $r_i$, or
2. $r_j\notin I(r_i[q])$ for $1\le q<\rho$: we have not encountered set $r_j$ in the index before while processing tokens at positions smaller than $\rho$.

The lookup returns the set of candidates *C*, which consists of set pairs with their similarity values and their end times. Considering only unseen sets avoids duplicating candidate pairs.

#### Example 8

Consider the lookup of set $r_6=\{c,d\}$ in the index in Fig. 6. While the list for token *c* is traversed, candidates $C=\{(r_6,r_5,0.33,11),(r_6,r_3,0.33,9)\}$ are collected; both $r_5$ and $r_3$ are unseen sets. The lookup of set $\{b,c\}$ in the same index returns three candidates: both sets from *I*(*b*) and one unseen set $r_5\in I(c)$.

**Index update.** The candidate index must be updated frequently, specifically when old sets expire and when new sets enter the sliding window. Before performing an index lookup, expired sets are removed from the candidate index in the $\textit{advance\_window}$ function (cf. Sect. 4).

The sets in the doubly-linked lists of the index are ordered by increasing end time. The list order is guaranteed as the timestamps of new sets cannot decrease. Therefore, expiring sets can be efficiently dequeued from the heads of the lists, and new sets are appended to the tails of the relevant lists. Overall, the index entries of a set *r* are inserted/deleted in *O*(|*r*|) time, independently of the index list lengths.

The candidate index stores all valid sets, i.e., the sets in sliding window *W*. A set *r* appears in |*r*| different index list entries, one for each token of *r*. A list entry is of constant size and stores the set identifier of *r* and its end time. Overall, the memory cost of the candidate index is $O(\sum _{r \in W}|r|)$ and depends only on the aggregated sets sizes in the sliding window.

#### Example 9

**Fig. 7:**
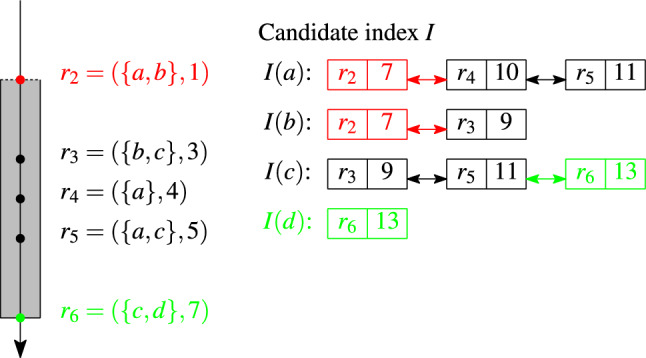
Index update: insertion and deletion

Figure 7 illustrates the index update for an expiring set $r_2$ (red elements) and a new set $r_6$ (green elements). The expiring set $r_2$ has two tokens which cause deleting the head-elements of two lists, *I*(*a*) and *I*(*b*). The new set $r_6$ adds one tail-element $(r_6,13)$ to the list *I*(*c*), and creates a new list *I*(*d*) with a single element $(r_6,13)$.

**Efficient index access.** A naive use of the candidate index offers little improvement over the baseline: only the set pairs with no overlap are avoided, while the use of the index tends to cause more cache misses than the baseline.

In the remaining sections, we will show how to use the index efficiently. When looking up a new set $r_i$, the index lists of its tokens are processed from tail to head. We introduce two new filter bounds to reduce the number of candidate pairs to form. The *positional upper bound* (Sect. 6.2) is the maximum similarity value that a new set can reach with any set in a given index list. Sets for which the upper bound is less than the minimum similarity required for a set pair to be relevant are skipped. The minimum required similarity value is defined by the *skyband lower bound* (Sect. 6.3). We discuss the overall candidate generation algorithm in Sect. 6.4 and show that the order in which the lists are processed can have a significant impact on performance (Sect. 6.5).

### Positional upper bound

Consider a lookup of set $r_i$ in the index *I*. The lookup returns a list *I*(*v*) for each token $v\in r_i$. Let $\rho$ be the token position in set $r_i$ that we examine. For the unseen sets $r_j\in I(r_i[\rho ])$ (i.e., sets which we have not encountered in the index before), we know that there are at least $\rho -1$ tokens in $r_i$ that do not exist in $r_j$. This information leads to the maximum possible similarity value between set $r_i$ and every unseen set $r_j\in I(r_i[\rho ])$ that only depends on the set $r_i$ and the token position $\rho$.

This principle has been used before in the context of specific set similarity functions (e.g., Jaccard [45]). Compared to previous work [48], (1) we do not assume a global order of tokens, and (2) we generalize the bound to the class of well-behaved set similarity functions.

#### Theorem 1

Given two sets *r*, *s*, and a well-behaved similarity function $\textit{set\_sim}(\cdot ,\cdot )$. If at least $\mu$ tokens of *r* do not exist in *s*, then the following upper bound on the similarity between *r* and *s* holds:

$$\begin{aligned} \textit{set\_sim}(r,s)= &   \textit{sim}(|r|,|s|,|r\cap s|)\\\le &   \textit{sim}(|r|,|r|-\mu ,|r|-\mu ) \end{aligned}$$

#### Proof

We show that $\textit{sim}(|r|,|s|,|r\cap s|)$ is maximized if $|s|=|r\cap s|=|r|-\mu$: We know that $|s|\ge |r\cap s|$ and $\mu$ tokens of *r* do not exist in *s*. Then, $|r|-\mu =|r\cap s|$ is the smallest length of *s* such that *r* and *s* have $\mu$ different tokens. Further, $|r|-\mu$ is the highest possible overlap $|r\cap s|$ since at least $\mu$ tokens of *r* do not exist in *s*. Thus, for $|s|=|r\cap s|=|r|-\mu$ the overlap is maximized and the set length of *s* is minimized. According to Definition 2 of well behaved set similarity functions, the similarity value increases with increasing overlap $|r\cap s|$ (Property 3) and decreasing set length |*s*| (Property 4). $\square$

Based on our observation and Theorem 1 we derive the following *positional upper bound*, $\textit{ub}(|r|,\rho )$, between set *r* and every unseen set in $I(r[\rho ])$.

$$\begin{aligned} \textit{ub}(|r|,\rho )=\textit{sim}(|r|,|r|-\rho +1,|r|-\rho +1) \end{aligned}$$

For any unseen set $s\in I(r[\rho ])$, $\textit{set\_sim}(r,s)\le ub(|r|,\rho )$.

#### Example 10

**Fig. 8:**
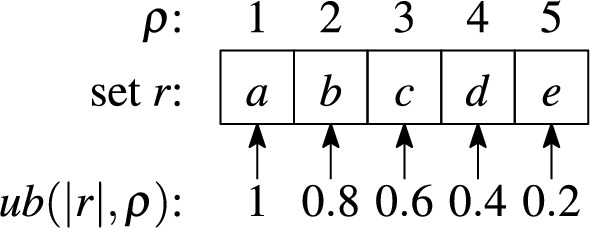
Example of the positional upper bound and Jaccard similarity

Fig. 8 illustrates the positional upper bound for the Jaccard similarity on set $r=\{a,b,c,d,e\}$ of length $|r|=5$. For example, the highest similarity value between set *r* and every unseen set in *I*(*d*) is 0.4.

### Skyband lower bound

The *skyband lower bound* is a filter derived from the pairs that are already in the stock. Intuitively, the skyband lower bound is the minimum similarity required for pair $(r_i,r_j)$ to be relevant. By integrating the skyband lower bound into the index loopkup, we can discard irrelevant pairs before inserting them into the stock.

A pair $$(r_i,r_j)$$ is *irrelevant*, if at any time in its validity interval $$[t_i,t_j+w)$$ at least *k* more similar pairs exist in the stock. An important observation is that if a pair is irrelevant at its end time, it is irrelevant in its entire validity interval. This is true because all pairs in the stock are valid at the join time, and a newly inserted pair can never increase the rank of any other pair in the stock. A pair may be irrelevant before it is inserted into the stock (then we can avoid inserting it), or it may become irrelevant due to the insertion of another pair. By inspecting the stock, we can derive the minimum similarity value for a pair to be relevant.

We define the *skyband lower bound*, *lb*(*t*, *k*), as the similarity of the *k*-th pair at time $t> t_J$ in stock *S*. The skyband lower bound marks the boundary of the *k-skyband* which is maintained in the stock (cf. Sect. 1). We use the skyband lower bound to detect irrelevant pairs. A pair $(r_i,r_j)$ with end time $t=t_j+w$ is irrelevant iff its similarity is below the lower bound at its end time *t*: $(r_i,r_j)\text { is irrelevant } \Leftrightarrow \textit{set\_sim}(r_i,r_j)< lb(t,k)$. For the efficient computation of *lb*(*t*, *k*) we refer to Sect. 7.3.

#### Example 11

**Fig. 9:**
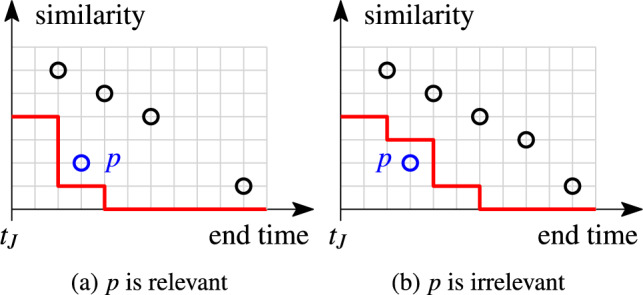
Skyband lower bound (red line) ($k=3$)

The red staircase functions in Fig. 9 show the skyband lower bound for two example stocks (black circle points) and $k=3$. We investigate the relevancy of the blue pair *p*. In Fig. 9a, *p* is relevant since the rank at its end time is $3\le k$. In Fig. 9b, we insert an additional pair into the stock. Then, *p* is irrelevant as the rank at its end time is $4>k$. New pairs cannot improve the rank of pairs that are already in the stock; at best, they leave it unchanged.

Together with the positional upper bound, the skyband lower bound allows us to stop processing an index list early. This happens when the minimum required similarity value of a pair to be relevant (skyband lower bound) exceeds the maximum similarity value that the pair can reach (positional upper bound). The following lemma shows that we converge to our stopping condition as we traverse the index lists in decreasing end time order.

#### Lemma 3

The skyband lower bound, *lb*(*t*, *k*), is a non-increasing function in *t*.

#### Proof

All pairs start at or before the join time. The *k*-th pair $p=(r_i,r_j)$ in the top-*k* list *T* at join time has similarity $\tau =\textit{set\_sim}(r_i,r_j)$ and end time $t=t_j+w$. When a pair $p\in T$ ends, a pair $p_i$ with similarity at most $\tau$ is promoted to position *k* in *T*. Thus, the skyband lower bound cannot increase. $\square$

### Efficient candidate generation algorithm

We use the positional upper bound and the skyband lower bound to efficiently prune candidates during the lookup in the candidate index *I*. More specifically, we employ the bounds as follows. For a new set $r_i$, we process the index list $I(r_i[\rho ])$ from tail to head, such that, the end times $t_j+w$ of the sets $r_j\in I(r_i[\rho ])$ do not increase (cf. Sect. 6.1). We form a candidate pair $(r_i,r_j)$ for each unseen set $r_j$. We stop processing the list as soon as the skyband lower bound exceeds the positional upper bound, i.e., $lb(t_j+w,k)>ub(|r_i|,\rho )$. This is correct due to Lemma 3: the lower bounds at the end times of all remaining unseen sets in the list will also exceed the upper bound threshold, i.e., no additional relevant pairs can be formed.

#### Example 12

**Fig. 10:**
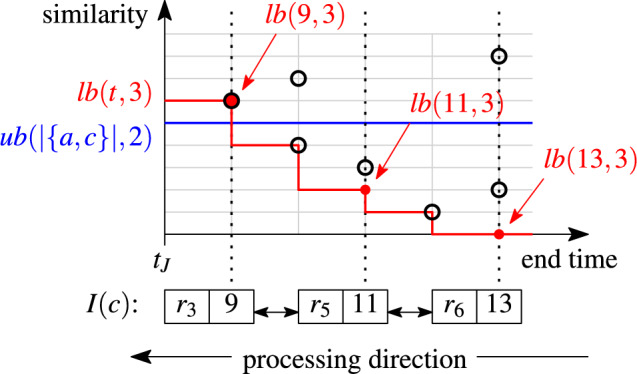
List processing with bounds, $k=3$

Consider the example in Fig. 10 and $k=3$. We look up the list *I*(*c*) for the second token *c* of set $r_i=\{a,c\}$. The list *I*(*c*) consists of three sets, $r_3=\{b,c\}$, $r_5=\{a,c\}$, $r_6=\{c,d\}$, ordered by their end times, $t_3+w=9$, $t_5+w=11$, $t_6+w=13$, respectively. The current pairs in the stock are marked with black circle points. The positional upper bound, $\textit{ub}(|r_i|,\rho )=\textit{ub}(|\{a,c\}|,2)=0.5$, is constant for the index list *I*(*c*) (blue line in the figure). The skyband lower bound, $\textit{lb}(t,k)=\textit{lb}(t,3)$ depends on the end times *t* (red staircase line). A set pair $(r_i,r_j)$, $r_j\in I(c)$, is relevant iff $r_j$ is unseen and the lower bound $\textit{lb}(t_j,3)$ is below the upper bound. We process the list *I*(*c*) from tail to head. Set $r_6=\{c,d\}$ is unseen. The lower bound at its end time is $\textit{lb}(13,3)=0$ and below the upper bound. Thus, the pair $(r_i,r_6)$ is relevant and returned as candidate. We continue with set $r_5=\{a,c\}$. This set has been processed earlier while traversing list *I*(*a*) for the first token *a* of $r_i$; therefore, the pair $(r_i,r_5)$ is not relevant. We have to check if any of the remaining sets in the list may be relevant. The lower bound at the end time of $r_5$ is $\textit{lb}(11,3)=0.2$ and below the upper bound. Therefore, we proceed to the unseen set $r_3$. For the pair $(r_i,r_3)$ to be relevant, its similarity must be at least $\textit{lb}(9,3)=0.6$, which is higher than the maximum similarity $\textit{ub}(|r_i|,\rho )=0.5$ that the pair can reach. Hence, $(r_i,r_3)$ is irrelevant and the stopping condition is triggered. If *I*(*c*) had more sets to be processed, none of them would lead to a relevant pair with the set $r_i$.

Algorithm 2 generates candidate pairs for a new set $r_i$ using the candidate index *I*. At this point, the index is up to date and the expired sets were removed in $\textit{advance\_window}$ (cf. Index update in Sect. 6.1). The candidate index is equipped with positional upper bound and skyband lower bound filters. For each token of the new set, $r_i[\rho ]$, we probe *I* to get a list $I(r_i[\rho ])$ of set IDs and their end times. The list is traversed from tail to head in line 5 until the stopping condition based on our upper and lower bound holds (line 7). The list elements, which form a relevant pair with set $r_i$, are stored with their lower bound values in hashmap *M*. Once all relevant sets are collected, we verify the pairs by computing their overlap to get the final set of candidates (lines 12–15). Finally, the new set $r_i$ is inserted into the index in line 16.

**Algorithm 2:**
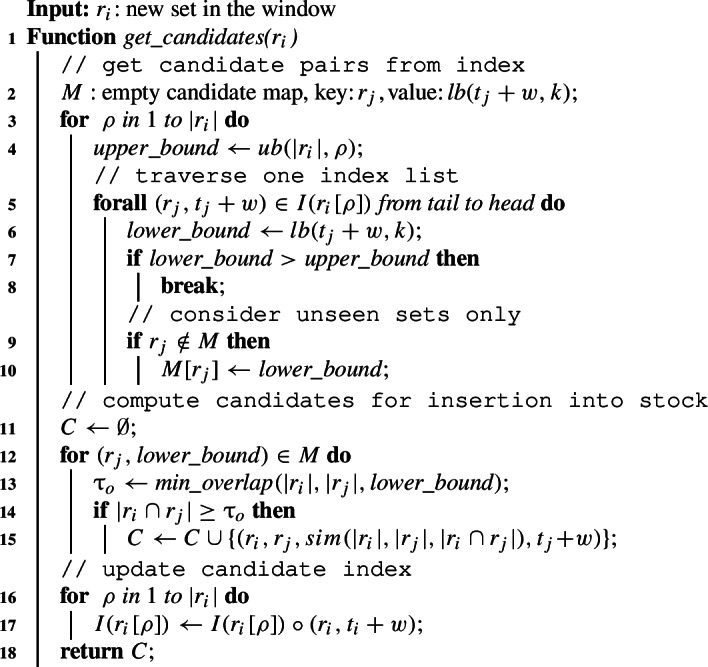
Efficient candidate generation.

A candidate pair $(r_i,r_j)$ is verified by checking $|r_i\cap r_j|\ge \uptau _o$, where $\uptau _o$ is the minimum overlap required such that the similarity between $r_i$ and $r_j$ is at least the skyband lower bound. The check is performed using the verification algorithm by Mann et al. [25], which scans the two sorted sets in a merge-like fashion, stopping early if $\uptau _o$ cannot be reached. Stopping early was shown to have a significant impact on the performance for threshold-based set similarity joins [25].

A specific set $r_j$ may appear in multiple lists. Since the value of $\textit{lower\_bound}$ for $r_j$ does not change during the execution of $\textit{get\_candidates}()$, we look up the bound in *M* and need not recompute it (line 6).

### Optimized token processing order

Before we process a new set $r_i$, we order its tokens. This is required for the merge-like set overlap computation. Algorithm 2 is correct for any token ordering. The order, however, impacts its performance.

A well-known approach is to order sets by decreasing token frequency, i.e., rare tokens appear earlier in the sorted sets. This is useful in two ways. First, the stop condition in the merge-like overlap computation improves with the number of mismatches, which are more likely for rare tokens. Second, rare tokens have short lists in the index. Processing short index lists first has a substantial impact on the performance. This is due to our upper bound, which improves with the lookup position of a token. A tighter upper bound allows us to skip a longer section of the index list. Thus, we want to process long lists as late as possible and use the bound to skip large fractions of the long lists.

#### Example 13

To illustrate this concept, consider looking up set *r* in Fig. 8 in the candidate index. The upper bound for the first token is 1, so the entire index list must be processed for the first token. The upper bound for the second token is 0.8 and we stop processing the corresponding index list as soon as the skyband lower bound exceeds 0.8. Since the upper bound decreases with the lookup position of the token, we can stop at smaller lower bounds for lists that we look up later. Stopping early has more effect on longer lists.

Non-streaming set similarity joins count the frequency of each token in a preprocessing step and establish the processing order of the index lists upfront. Token frequencies ensure that the shortest list is processed first. This is not possible in our setting since the sets arrive on a stream and are not known upfront. An ordering heuristic for streams works as follows: Each token is numbered when it first appears in the stream. Then, a new set is sorted in descending order of the first occurrence of its tokens, i.e., tokens that occurred later in the stream are sorted earlier in the set. The idea is that frequent tokens are more likely to occur earlier in the stream than infrequent ones.

In our experiments, we show that this ordering heuristic is effective if the token distribution is stable over time, i.e., a token appears with the same probability in each subsection of the stream. Unfortunately, some real world data does not satisfy this assumption, which leads to inefficiencies. To deal with skewed token distributions, we process a new set $r_i$ as follows. We first retrieve the index lists of all tokens of $r_i$ and heapify the lists such that the shortest list is on top of the heap. We then pop the lists and process them until the heap is empty. This approach substitutes the order in line 3 of Algorithm 2.

The merge-like overlap computation between a new set $r_i$ and its candidates $r_j$ requires that the sets are sorted in the same order. To avoid sorting all candidate sets for each new set $r_i$, we do not use the list processing order for the overlap computation. Instead, we use the same global order for all sets, so that each set is sorted only once.

## Maintaining the join result

Stock *S* maintains the join result. This includes ranking the *k* most similar set pairs at the join time $t_J$, and keeping enough valid replacements for the expiring result pairs that leave the sliding window. We define the following functionalities of the stock.

- $\textit{topk}()$: Return the top-*k* result at the join time $t_J$.
- $\textit{advance\_window}(t)$: Increase the join time $t_J$ to *t*. Remove expired sets from the window and add new sets to the window. Remove invalid pairs from the stock and from the *end time index*, which is introduced in this section. Delete expired sets from the candidate index.
- $\textit{lb}(t,k)$: Get the skyband lower bound at time *t*, i.e., the similarity of the *k*-th pair at time $t> t_J$. The skyband set pairs are maintained in the stock.
- $\textit{insert\_to\_stock}(C)$: Insert a collection of candidate pairs *C*, which all start at join time $t_J$, into the stock.

In this section, we design efficient solutions for the stock functionalities. We first introduce the stock data structure and show how it is leveraged to compute the skyband lower bound and the insertion to stock (Sect. 7.1). The functions $\textit{advance\_window}(t)$ and $\textit{lb}(t,k)$ are discussed in Sects. 7.2 and 7.3, respectively, before introducing the efficient *merge insert* algorithm(Sect. 7.6) which improves over the simpler *sweep line insert* (Sect. 7.4) and *cleanup insert* techniques (Sect. 7.5).

### Stock data structure

For a pair $(r_i,r_j)$, the stocks stores a quadruple $p=(r_i,r_j,\textit{sim}(r_i,r_j),t_j+w)$. We refer to the elements of the stock simply as *pairs*. We use $\textit{sim}(p)=\textit{sim}(r_i,r_j)$ to denote the similarity of the pair $(r_i,r_j)$ and $\textit{end}(p)=t_j+w$ to denote the pair’s end time. *S* is implemented as a binary search tree ordered by decreasing similarity (and lexicographically by descending end time, ascending *i* and *j* to break ties). Therefore, $\textit{topk}()$ is trivial and traverses the first *k* elements of *S* in sort order.

**Search and update.** The stock data structure *S* supports the five search and update operations listed in Table 4. All operations can be executed in $O(\log |S|)$ time. For implementation details we refer to Sect. 8.

**Table 4:** Operations supported by the stock data structure

Operation	Explanation
$S.\textit{at}(i)$, short *S*[*i*]	Return the *i*-th element of *S* in the sort order
$S.\textit{search}(s)$	Return $p\in S$ with the lowest similarity $\ge s$
$S.\textit{rank}(p)$	Return the rank of *p* in the sort order of *S*
$S.\textit{remove}(i)$	Remove the *i*-th element of *S* in the sort order
$S.\textit{insert}(p)$	Insert *p* into *S*

**Minimal stock.** We call stock $S$ *correct* if it contains all pairs that may be required in the future to maintain the top-*k* join result *T*, i.e., all pairs that are relevant at join time $t_J$ (cf. Sect. 6.3). We call $S$ *minimal* if it is correct, and removing any of the pairs will render the stock incorrect. The stock maintained by the baseline (cf. Sect. 4), which is correct but not minimal, is quadratic in the window size |*W*|. The size of the minimal stock is linear in |*W*|.

#### Lemma 4

The size of a minimal stock $S$ is $O(k\cdot |W|)$.

#### Proof

**Fig. 11:**
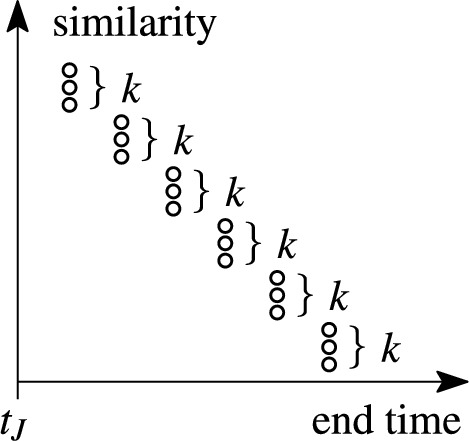
Stock worst case for $k=3$

Each pair in the minimal stock (cf. *minimal stock* in Sect. 1) is dominated by at most $k-1$ other pairs, i.e., pairs with higher similarity and end time. In the worst case, each pair is dominated by a different set of $k-1$ other pairs. In window *W*, each set may have a different timestamp, leading to |*W*| different possible end times of set pairs in the stock. Each of such |*W*| set pairs can be dominated by at most $k-1$ other pairs. Hence, $|S|=O(|W|+|W|\cdot (k-1))=O(k\cdot |W|)$. Figure 11 visualizes the worst case. $\square$

**End Time Index.** Additionally to the stock, we introduce the *end time index* *E* that maintains the same elements as the stock $S$, but orders them by ascending end time (ascending similarity, descending *i*, *j* for pairs $(r_i,r_j)$). Similarly to $S$, *E* is implemented as a binary search tree that supports search and update operations from Table 4 in logarithmic time. Index *E* is updated whenever $S$ is updated, thus $|E|=|S|$.

The following theorem establishes a connection between the end time index *E* and the stock *S* that is leveraged for the efficient computation of the skyband lower bound and incremental stock update.

#### Theorem 2

Let $t\ge t_J$ be a timestamp, $p=E.\textit{search}(t)$ the pair in *E* with the smallest end time $\textit{end}(p)\ge t$, and $v=E.\textit{rank}(p)$ the rank of *p* in the end time index *E*. If stock *S* is minimal, then the *k*-th pair in *S* at time *t* is $S[k+v-1]$.

#### Proof

By induction on *v*. *Base case.* $p_1=E[1]$ is the first pair to end in the stock *S*. Until the end time of $p_1$, i.e., at any time $t_1\in [t_J,\textit{end}(p_1))$, the *k*-th pair in the stock is *S*[*k*]. This holds because one of the first *k* pairs in the stock must end first. Otherwise, there would be another pair $p'\in S$ at rank greater than *k*, which ends first. This, however, is not possible in a minimal stock because $p'$ would be dominated by the first *k* pairs in the stock.

*Inductive step.* We assume that the *k*-th pair in the stock at any time $t_v\in [\textit{end}(p_{v-1}),\textit{end}(p_v))$, i.e., the time interval depicted by the *v*-th pair to end, is $S[k+v-1]$. For $v+1$, we consider two cases. *Case 1: unique end times in E*. The pair $E[v+1]$ defines the next interval $[\textit{end}(p_{v}),\textit{end}(p_{v+1}))$. Since the pair $S[k+v-1]$ is now invalid, the next element in the stock, $S[k+v]$, is promoted to become the *k*-th pair in *S*. *Case 2:* *n* *entries in E* *with the same end time.* *v* is always the position of the first of these entries in *E*. The pair $E[v+n]$ defines the next interval, invalidating the former pairs *E*[*v*] to $E[v+n-1]$ and promoting $S[k+v-1+n]$ to rank *k* in *S*. $\square$

#### Example 14

**Fig. 12:**
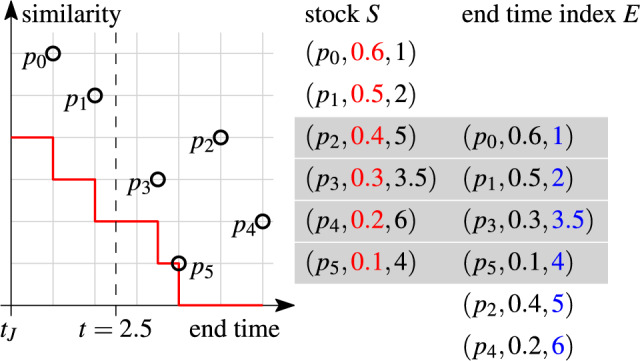
Alignment of the stock *S* and the end time index *E* as a result of Theorem 2 ($k=3$)

Fig. 12 shows six pairs $p_0,\ldots ,p_5$, stock *S*, end time index *E*, and the skyband lower bound for $k=3$ (red line). *S* and *E* store the same elements ordered by similarity (red values) resp. end time (blue values). The pairs in *S* are aligned with the pairs in the plot. The end time values are relative to the join time $t_J$. We shift the orders by $k-1$ positions such that *E*[*v*] is aligned with $S[k+v-1]$ (gray bars). Note that the pairs in the bars define the steps of the skyband lower bound, e.g., the first bar defines the point (0.4, 1), where the first step ends. This is the result of Theorem 2 and holds if the stock is minimal.

### Removing invalid pairs efficiently

The $\textit{advance\_window}(t)$ operation, among other tasks (cf. beginning of this section), removes all invalid pairs *p* from the stock $S$ and the end time index *E* with the end time $\textit{end}(p)<t$. A naive solution scans $S$, checks the end time of each pair, and removes expired pairs. For $n\le |S|$ expired pairs, the runtime is $O(|S|+n\log |S|)$. This is too slow as the window is advanced with each new set in the stream.

Our implementation of $\textit{advance\_window}(t)$ uses the end time index and scans it as long as $\textit{end}(p)<t$. Then the scan stops, and the remaining pairs are not touched. Each scanned pair is removed from both the stock and the end time index. The removal of $n\le |S|$ invalid pairs takes $O(n\log |S|)$ time. Since each pair can be removed only once, the worst case $n=|S|$ is infrequent, and the average complexity is $O(\log |S|)$. The same complexity holds for the end time index.

### Efficient lower bound computation

The skyband lower bound *lb*(*t*, *k*) (cf. Sect. 6.3) is the similarity of the *k*-th pair in the stock *S* at some time $t>t_J$. It is used during candidate generation and is evaluated for each entry in the index lists until the stopping condition is reached.

A straightforward implementation scans the stock *S* and returns the *k*-th pair *p* that satisfies $\textit{end}(p)\ge t$. This takes *O*(|*S*|) time, which is too expensive since the lower bound needs to be computed for each candidate pair. We leverage Theorem 2 and use the end time index *E* to retrieve the *k*-th pair at time *t*, Algorithm 3.

**Algorithm 3:**
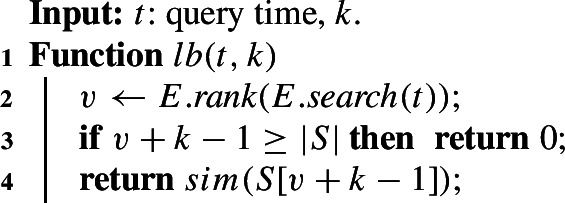
Skyband lower bound at time *t* for given *k*.

To compute *lb*(*t*, *k*), we search *E* for the first pair (in *E*’s sort order) with the end time greater or equal *t* and retrieve its rank *v* (line 2). Operation *lb*(*t*, *k*) is the similarity of the pair at position $v+k-1$ in *S* (line 4). All these operations are logarithmic in $|S|=|E|$.

#### Example 15

We compute *lb*(*t*, *k*) for $t=2.5$ and $k=3$ in Fig. 12. $p_3$ at position $v=3$ is the first pair in *E*’s sort order with end time $\ge t$. The aligned pair $S[v+k-1]=S[5]=p_4$ has similarity 0.2, which is the skyband lower bound at time $t=2.5$.

### Sweep line insert

The $\textit{insert\_to\_stock}(C)$ operation adds a set of candidate pairs, *C*, into the stock. The challenge is to keep the stock minimal. New pairs may turn out to be irrelevant (in which case they should not be inserted), or they may render other pairs irrelevant (which then must be removed).

#### Example 16

**Fig. 13:**
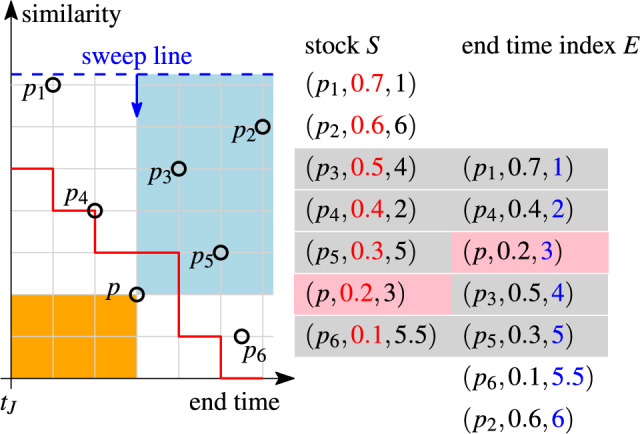
Relevance check using a sweep line, $k=3$. Disrupted alignment of stock and end time index after inserting an irrelevant pair

Assume we want to insert pair *p* into the stock in Fig. 13. To check if *p* is relevant, the rank at its end time $\textit{end}(p)$ must be at most *k*. The rank of *p* is determined by the number of stock elements $p'$ that do not end before *p* and are at least as similar, i.e., $\textit{end}(p)\le \textit{end}(p')$, $\textit{sim}(p)\le \textit{sim}(p')$. There are three such pairs ($p_2,p_3,p_5$, blue area); thus, *p* is irrelevant (rank $4>k$ at end time).

Note that inserting the irrelevant pair *p* causes the stock to no longer be minimal. As a consequence, the alignment of *S* and *E* (gray horizontal bars in Fig. 13) stated in Theorem 2 is disrupted.

Let $p\in C$ be the candidate pair to be inserted. Our Sweep Line Insert algorithm starts with verifying the relevance of *p*. It uses a sweep line to scan *S* in sort order, and it counts all pairs $p'\in S$, $\textit{end}(p)\le \textit{end}(p')$, $\textit{sim}(p)\le \textit{sim}(p')$ (blue area in Fig. 13). If *p* is irrelevant, it is rejected. Otherwise, *p* is inserted. Since inserting *p* may cause other pairs to become irrelevant, they must be checked too. The Sweep Line Insert algorithm is executed for each pair $p''$, such that, $\textit{end}(p)\ge \textit{end}(p'')$, $\textit{sim}(p) \ge \textit{sim}(p'')$ (orange area in Fig. 13). Thus, the overall runtime of Sweep Line Insert is $O(|C||S|^2)$ for |*C*| candidate pairs.

The bottleneck of this solution is that each candidate pair is treated separately and the irrelevant pairs are removed one by one. We observe that all irrelevant pairs can be removed from the stock by scanning it only once which we leverage in the Cleanup Insert algorithm.

### Cleanup insert

We propose the Cleanup Insert algorithm that (1) adds all candidates *C* to the stock without any relevance checks, and (2) removes all irrelevant pairs in a single pass over the stock. This is a major improvement over the sweep line algorithm that requires a quadratic number of operations in the stock size |*S*| for each candidate pair.

First, we present a *cleanup* algorithm that uses the end time index *E* to remove all *i* irrelevant pairs from stock *S* in time $O(|S| + i \log |S|)$. Second, we optimize cleanup for the use with insert. We leverage the following property of non-minimal stocks.

#### Lemma 5

Let *e* be the position of the first irrelevant pair *p* in *E*, $p=E[e]$. Then, the position *s* of *p* in $S$, $p=S[s]$, exceeds $e+k-1$, i.e, $s>e+k-1$.

#### Proof

$p=E[e]$ is the first irrelevant pair in *E*. (1) There must be at least *k* pairs that dominate *p*, otherwise *p* would be relevant. (2) There are $e-1$ pairs in the stock that end before *p*. These pairs must have similarity higher than *p*. Otherwise, they all would be dominated by *p* itself and the same pairs which dominate *p*. By (1) and (2), there are at least $k+e-1$ pairs before *p* in *S*. $\square$

After inserting all candidates *C*, we use Lemma 5 to clean the stock as follows. We scan the end time index *E* (in its order) and for each position *e* we verify if the rank of pair *E*[*e*] in stock *S* exceeds $e+k-1$. In such a case, the pair is irrelevant and is removed from both the end time index and the stock. We repeat the procedure from position *e* until all pairs in *E* are processed. Computing the rank of pair *E*[*e*] in *S*, $S.\textit{rank}(E[e])$, requires $O(\log |S|)$ time.

We avoid the logarithmic factor in our cleanup algorithm (Algorithm 4 without gray-shaded parts) as follows. Let $\prec _S$ be the order relation of the elements in the stock *S* (cf. Sect. 7.1). We start with $e=1$ and iterate through the pairs *E*[*e*] and *S*[*s*] simultaneously such that $s=e+k-1$. If $S[s]\prec _S E[e]$, then the rank of *E*[*e*] in *S* is above $e+k-1$, and *E*[*e*] is irrelevant. Thus we avoid computing the exact rank of *E*[*e*] in *S*. The order relation $\prec _S$ can be evaluated in constant time. Thus, complexity of removing *i* irrelevant pairs is $O(|S|+i\log |S|)$.

#### Example 17

We clean the stock in Fig. 13, $k=3$. Initially, $e=1$ and $s=e+k-1=3$ (topmost gray bar). $S[3]=p_3\nprec _S p_1=E[1]$ and thus $p_1$ is relevant. In the next step $e=2$. Then, $S[4]=p_4=E[2]$ and $p_4$ is relevant. For $e=3$, $S[5]=p_5\prec _S p=E[3]$. Hence, *p* is irrelevant and is removed. We proceed until $s=|S|$.

**Optimized cleanup.** The cleanup can be optimized for insertion by scanning only the regions of *S* that may contain irrelevant pairs. We identify these regions by inspecting the set of inserted candidate pairs *C*.

#### Theorem 3

Let stock *S* be minimal, *C* the set of candidate pairs to insert into stock, $\max _{\textit{sim}}=\max _{c\in C}\{\textit{sim}(c)\}$ and $\max _{\textit{end}}=\max _{c\in C}\{\textit{end}(c)\}$ the maximum similarity resp. end time of all pairs $c\in C$. After inserting candidates from *C* into *S* (without removing irrelevant pairs), the following holds for all pairs $p\in S\cup C$: if *p* is irrelevant, then $\textit{sim}(p)\le \max _{\textit{sim}}$ and $\textit{end}(p)\le \max _{\textit{end}}$.

#### Proof

Case (1): $p\in C$. The proof is straightforward. Case (2): $p\in S$. For a pair $p\in S$ to become irrelevant, one of the inserted pairs from *C* must dominate *p*, i.e., $\exists _{c\in C}: \textit{sim}(c)>\textit{sim}(p)\wedge \textit{end}(c)>\textit{end}(p)$. However, $\forall _{c\in C} \max _{\textit{sim}}\ge \textit{sim}(c) \wedge \max _{\textit{end}}\ge \textit{end}(c)$. Thus, *p* is irrelevant $\Rightarrow$ $\max _{\textit{sim}}>\textit{sim}(p)\wedge \max _{\textit{end}}>\textit{end}(p)$. $\square$

The optimized version of Cleanup Insert (Algorithm 4 including gray-shaded parts) uses Theorem 3 to scan only those parts of the stock *S* and the end time index *E* that might store irrelevant pairs.

#### Example 18

As an example, consider the stock in Fig. 13 and assume that the candidates $C=\{p_4,p_5\}$ have been inserted. With $\max _{\textit{sim}}=\textit{sim}(p_4)=0.4$ and $\max _{\textit{end}}=\textit{end}(p_5)=5$ we only need to evaluate four elements of *S* and *E*. The algorithm starts the scan at $s=4$ in *S* (since $p_4=S[4]$) and $e=s-k+1=2$ in *E*, and ends after four iterations. (1) $s=4$, $e=2$, $S[4]=p_4\nprec _S p_4=E[2]$. (2) $s=5$, $e=3$, $S[5]=p_5 \prec _S p=E[3]$; $S.\textit{remove}(S.\textit{rank}(p)=6)$; $E.\textit{remove}(3)$. (3) $s=5$, $e=3$, $S[5]=p_5\nprec _S p_3=E[3]$. (4) $s=6$, $e=4$, $S[6]=p_6\nprec _S p_5=E[4]$. The algorithm terminates with $s=7>|S|=6$.


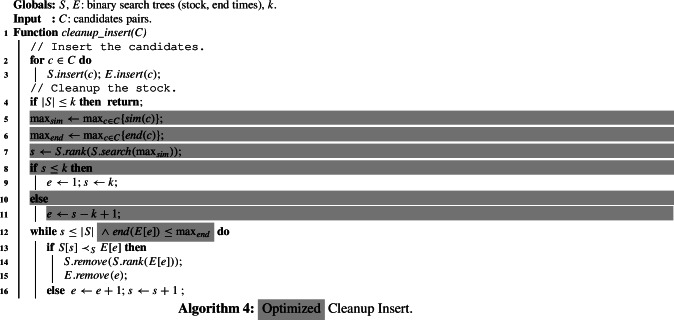


### Merge insert

We present our Merge Insert algorithm, which leverages the pruning power of the skyband lower bound to insert only relevant pairs and the optimized cleanup technique to keep the stock minimal. Merge Insert requires only a single scan of the stock and the candidates. Intuitively, inserting candidates and cleaning the stock are interleaved in a merge-like approach.

**Fig. 14:**
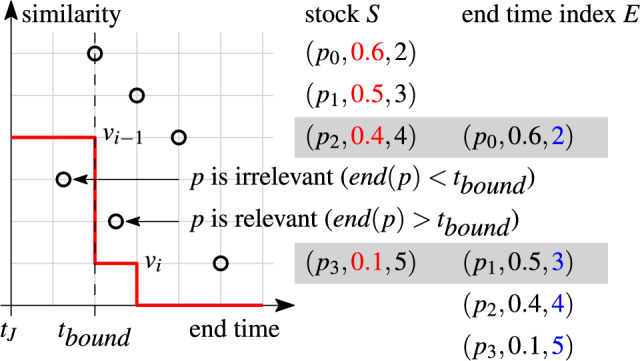
To insert or not to insert

**Fig. 15:**
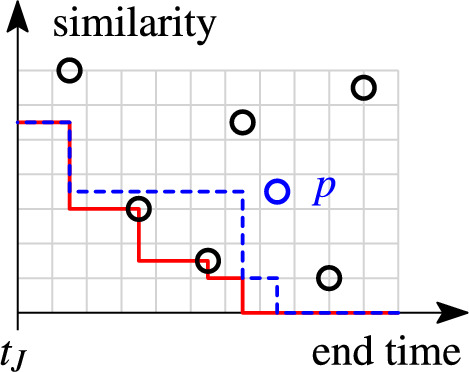
Inserting pair *p* causes removal of two pairs

**Overview.** The Merge Insert algorithm, Algorithm 5, processes both the stock items and the candidates in the order of the stock elements (descending similarity), and a merge-like approach is used to verify candidate pairs before they are inserted. Consider Fig. 14. Intuitively, we walk along the skyband lower bound (red line; cf. Sect. 6.3) following the alignment of stock and end time index (gray boxes; cf. Theorem 3). Assume the current vertex of the skyband lower bound is $v_i$. When we insert the candidates with similarity between that of the vertices $v_{i-1}$ and $v_{i}$, their end times must be above $t_{\textit{bound}}$, i.e., the end time of vertex $v_{i-1}$. Irrelevant candidates are never inserted, but the insertion of relevant candidate pairs may render other pairs irrelevant. In Fig. 15, we insert a new pair *p* (blue) into the stock. *p* causes the skyband lower bound to change (old: red solid, new: blue dashed) and hence two pairs from the stock are no longer relevant. Since irrelevant pairs can only appear after the current position in the stock, they will be removed as we proceed (like in the cleanup algorithm).

**Special case.** Algorithm 5 deals with the special case when the stock has less elements than *k* in lines 2–9. *i* (line 2) is the number of candidates that fit in the stock before its size exceeds *k*. We insert the first $k-|S|$ pairs from *C* (with decreasing similarity). If $|C|\le k-|S|$, all |*C*| pairs are inserted and the algorithm terminates.

**Initialization.** Lines 10–19 initialize the end time threshold $t_{\textit{bound}}$ and, similarly to the cleanup insert algorithm, indices *s* and *e*. $t_{\textit{bound}}$ is utilized to prune irrelevant candidates where the end time is not sufficiently high. *s* is the rank of the first candidate in the stock $S\cup \{c_i\}$. *e* is aligned such that (*E*[*e*], *S*[*s*]) defines a skyband lower bound vertex. If the resulting *s* is smaller than *k*, *s* is initialized to *k* and *e* to 1 (first step on the skyband lower bound).

**Main loop: insert and clean up.** In lines 20–32 the algorithm iterates over the stock *S* and the end time index *E* in a merge-like fashion. Intuitively, the main while loop (line 20) iterates over the steps of the skyband lower bound doing one step at a time (cf. Fig. 14). Changes to the skyband lower bound caused by insertions are incorporated in this loop such that the steps of the skyband lower bound are updated while iterating (cf. Fig. 15). The main loop exits when either the entire stock or all the candidates are scanned.

Every iteration of the inner while loop (lines 21–25) has two objectives. (1) Find the first relevant candidate that is more similar than *S*[*s*], and insert it into *S* and *E*. A candidate $c_i$ is inserted at position *s*, so $c_i$ becomes *S*[*s*], and the loop exits after the first insertion (as $\textit{sim}(c_{i+1})\le \textit{sim}(c_i)=sim(S[s])$). (2) While searching for the relevant candidate, prune all irrelevant ones using the end time threshold $t_{bound}$ as illustrated in Fig. 14.

The main loop proceeds like the cleanup algorithm (lines 26–32), except that also $t_{bound}$ is updated. If inserting a candidate causes stock pairs irrelevant, they are removed as in the cleanup algorithm by evaluating the alignment of *S* and *E* and using Theorem 5.

**Remaining candidates.** After scanning the entire stock, i.e. reaching the bottom of the skyband lower bound, there may still be candidates left. This is the case for the candidate pairs that are less similar than the least similar pair in *S*. Lines 33–38 process the remaining candidate pairs. Those of them that are irrelevant are pruned using the last step in the skyband lower bound as a threshold, i.e., $\textit{end}(E[e-1])$. Similarly to the main loop, inserting a candidate changes the last step of the skyband lower bound, and thus the relevancy threshold is increased to the end time of the inserted pair. 
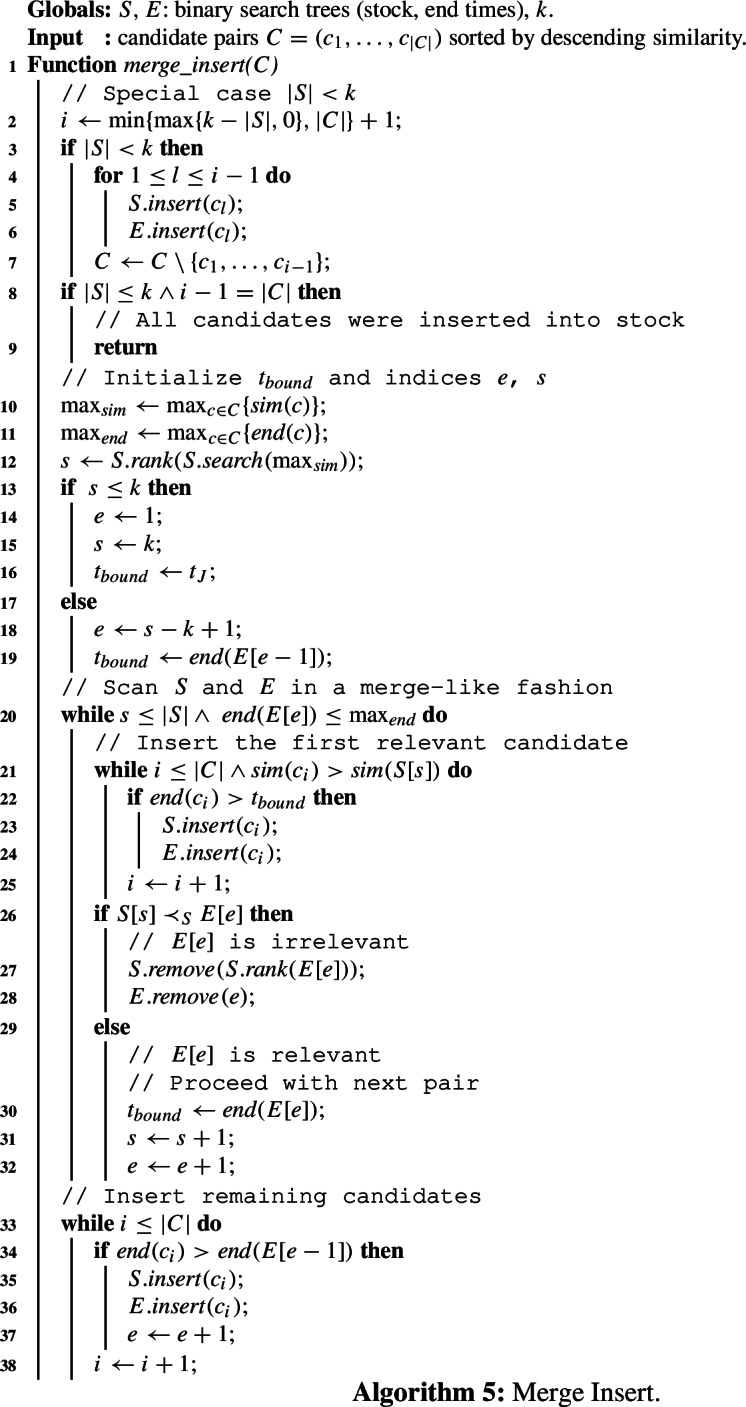


**Complexity.** The complexity of merge insert depends on the sizes of $S$ and *C*. Despite scanning only the relevant region of the stock (cf. Theorem 3), potentially each candidate pair has to be inserted, and each pair from the stock has to be removed. Inserting or deleting a pair takes $O(\log |S|)$ yielding a worst-case complexity of $O((|S|+|C|)\log (|S|+|C|))$.

## Experiments

**Algorithms.** We compare SWOOP with the following algorithms:

- SCase: State of the art for top-*k* joins over streams [37].
- Static: State of the art for top-*k* joins on static collections of sets [45]. We adapt this algorithm to streams, such that, whenever a new set arrives, we execute the top-*k* set similarity join algorithm, and compute the top-*k* from scratch.
- Base: Baseline algorithm as presented in Sect. 4.

**Datasets** In our empirical evaluation, we use five data streams with different characteristics. Table 5 shows the stream length (number of sets), the average set size, and the size of the token universe (number of distinct tokens) for each of the streams.

TWEET. Geocoded tweets collected at Daisy^2^ from February to April 2017. A tweet is a set of words with the posting time as a timestamp.

DBLP. Articles from DBLP^3^ [22]. A set is a publication and the tokens correspond to the words in the authors and title fields. The timestamp is the modification date from DBLP’s XML file.

FLICKR. Photo meta-data.^4^ A set consists of tokens from the tag or title text describing a photo. The timestamps are artificial and assigned uniformly at random between 0 and 10,000 s.

ENRON. E-mail data. A set is formed by the words in the subject and body fields, and the timestamp is defined by the send time.

INDUSTRY. Workflow instances from an ERP system. A set consists of pairs of subsequent workflow activities, and the timestamp is that of the last activity in the workflow. The timestamps were scrambled by the data provider for anonymization purposes.

**Table 5:** Dataset statistics

Dataset	Stream length	Avg. set size	Universe size
TWEET	$3.4 \cdot 10^7$	13.44	$3.7\cdot 10^7$
FLICKR	$1.2 \cdot 10^6$	10.05	$8.1\cdot 10^5$
DBLP	$5.5\cdot 10^6$	12.10	$1.7\cdot 10^6$
ENRON	$2.5\cdot 10^5$	302.2	$7.3\cdot 10^5$
INDUSTRY	$4.9\cdot 10^7$	13.07	$1.1\cdot 10^4$

**Measures.** We analyze the performance of the algorithms using the following measures.

*Average window size* $\overline{|W|}$ is the average number of sets in the sliding window *W* for a given time duration *w* of the window, assuming that the sets in the stream arrive at a constant rate. The conversion factor between the average window size $\overline{|W|}$ and its duration *w* is computed as $\frac{t_{\max }-t_{\min }}{|R|}$, where $t_{\min }$ and $t_{\max }$ are the minimum and maximum timestamps of the stream, respectively, and |*R*| is the number of records. The shortest and the longest window durations for each dataset and their conversion to the average window size $\overline{|W|}$ are shown in Table 6.

*Pre-candidates* are the set pairs that must be formed when a new set arrives in the stream. In Base and SCase, a new set will form a pre-candidate with each set in the sliding window. In SWOOP and Static, the number of pre-candidates is the number of processed index list items.

*Candidates* are the pre-candidates that are sent to the stock for insertion. Base sends all pre-candidates (with similarity larger than zero) to the stock. SWOOP and SCase filter the pre-candidates using a lower bound. Static does not use a stock and recomputes the join result for each window position.

*Set rate* is the average number of processed sets per second and thus measures the performance of an algorithm. We map string tokens to integers as discussed in Sect. 6.5. This process is identical for all algorithms and is not considered in the set rate.

*Latency* is the time difference between the appearance of a set in the stream and the update to the top-*k* result. It includes candidate generation, stock update, and potential waiting times in the input queue before the set can be processed.

**Table 6:**
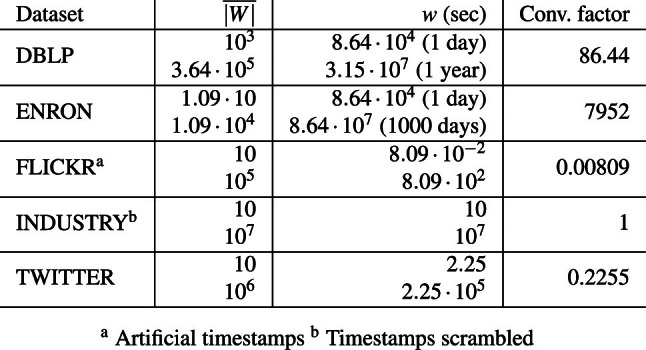
Minimum and maximum average window size $\overline{|W|}$, window duration *w*, and conversion factors

**Experimental environment.** We conduct the experiments on an 8-core Intel Xeon E5-2630 v3 CPUs with 2.4 Ghz, 96 GB of RAM, and 20 MB cache (shared across cores), running Debian 9. Our code is written in C++ and is compiled with GCC using the -O3 option. We measure the peak heap memory using the libmemusage library.

**Implementation details.** The source code and the datasets are available online [1]. We implemented all algorithms in C++ using data structures that are available from STL and Boost.^5^ For the binary search trees $S$ and *E* in SWOOP, we use the Boost Multiindex container. We define one Multiindex structure that stores the stock $S$ and provide two indices (for $S$ and *E*) on this container.

### Scalability

We evaluate the scalability of SWOOP and its competitors. We vary the average window size and the result size *k*, and we use all datasets. Missing values for an algorithm indicate that the stream could not be processed within 20k seconds (FLICKR, ENRON) resp. 200k seconds (other datasets).

**Fig. 16:**
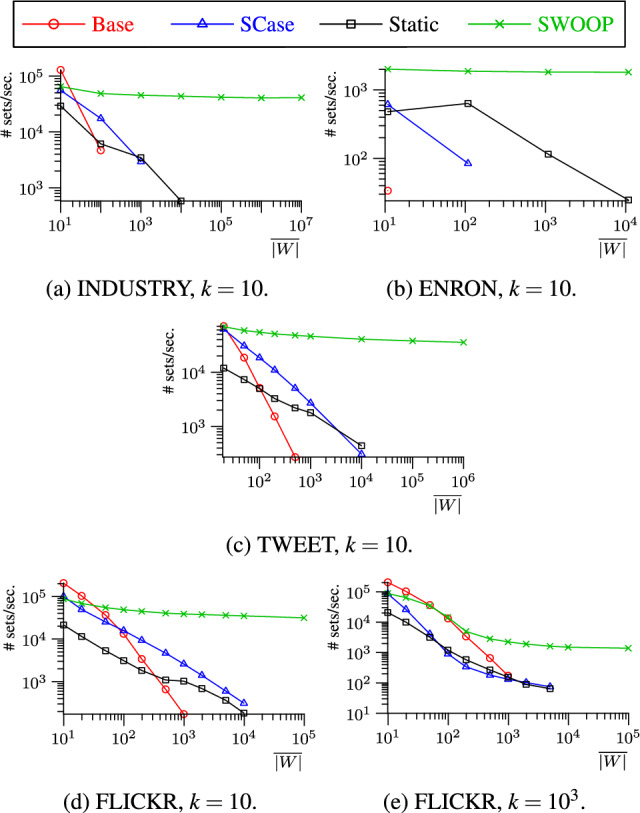
Set rate for different datasets

**Scalability in the window size.** We measure the set rate for different window sizes $\overline{|W|}$. Figure 16 shows the results. For a small window size close to *k*, even Base performs well. For larger windows, however, the set rates of Base, SCase, and Static decrease sharply. When we increase the window size by a factor of 10, the set rate of SCase decreases over all datasets by a factor of 3.1–8.7, the set rate of Base by a factor of 15–76, the set rate of Static by a factor of up to 6.7. SWOOP clearly outperforms all other approaches and scales well with the window size. In fact, for $k=10$ the performance between $\overline{|W|}=10^2$ and the largest window tested on the respective dataset decreased by less than a factor two. For a larger result size of $k=10^3$, we observe a similar behavior starting with $\overline{|W|}=10^3$.

**Fig. 17:**
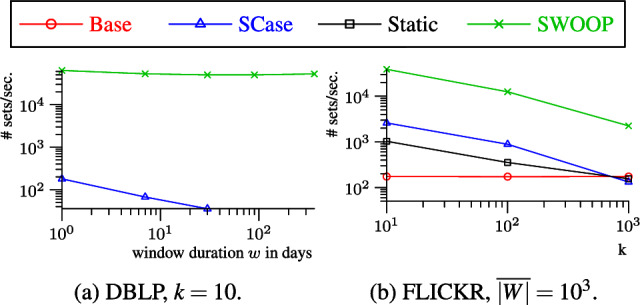
Set rate for varying window duration *w* (**a**) and result size *k* (**b**)

The DBLP stream is particularly challenging due to its skewed distribution of the timestamps. Figure 17a shows the results for varying window durations *w* (the average window size is not meaningful for DBLP since it is heavily skewed). Base and Static run into a timeout even for the smallest window duration of $w=1$ day. SCase is slower than SWOOP by two to three orders of magnitude, and only SWOOP is capable of processing the DBLP stream for all window sizes without timeouts. The set rate of SWOOP is affected little by the window size. For more details on DBLP dataset refer to Sect. 8.4.

**Scalability in k.** In Fig. 17b, we vary the result size *k* for a fixed average window size $\overline{|W|}=10^3$ on the FLICKR stream. This stream can be processed by algorithms for $k=10$. The set rate of Base is low, but does not depend on *k*. This is because Base does not leverage lower *k* values to decrease the stock size or reduce the number of candidates. SCase, Static, and SWOOP run faster for smaller *k* values. SWOOP is consistently faster than SCase and Static by more than one order of magnitude.

### Performance analysis

We analyze the performance advantage of SWOOP over its competitors in detail.

**Fig. 18:**
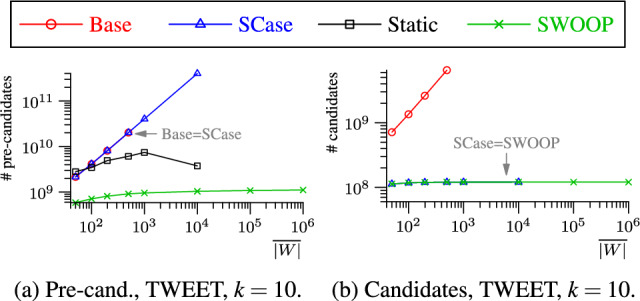
Performance in the number of pre-candidates (**a**) and candidates (**b**)

**Pre-candidates.** Figure 18a shows the number of pre-candidates (the sum over all windows) on the TWEET stream. Base and SCase form a pre-candidate with each set in the sliding window, which leads to a large number of pre-candidates. SWOOP uses the candidate index to reduce the number of pre-candidates that must be considered. The candidate index is highly effective. SWOOP considers only a small fraction of the pairs that its competitors must process, and the number of pre-candidates grows slowly with the window size. This explains SWOOP’s scalability to large windows.

**Candidates.** In Fig. 18b we measure the number of candidates (the sum over all windows). Base cannot prune any candidates, and all candidates are added to the stock.^6^ SCase and SWOOP both insert the same pairs into the stock, so the number of candidates is the same. While SCase recomputes the stock from scratch for each new set in the stream, SWOOP updates the stock incrementally.

**Fig. 19:**
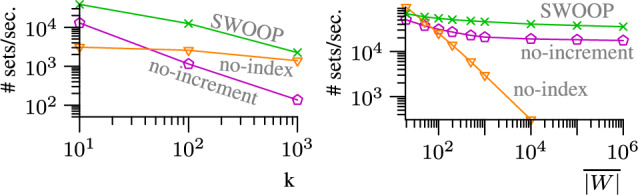
SWOOP without a candidate index (no-index) and without incremental stock maintenance (no-increment)

**Stock maintenance and candidate index.** In Fig. 19, we evaluate the effect of the incremental stock maintenance and the candidate index on the overall performance of SWOOP. To this end, we implement a version of SWOOP without candidate index (labeled *no-index*) and another version that recomputes the stock from scratch like SCase, i.e., it does not support incremental updates (labeled *no-increment*).

Clearly, both the candidate index and the incremental stock maintenance contribute to the performance of SWOOP. As *k* increases, the skyband lower bound becomes looser, leading to more pre-candidates and reducing the effectiveness of the candidate index (cf. Fig. 19a). The incremental stock maintenance of SWOOP gains more for larger values of *k* and outperforms the no-increment variant by up to an order of magnitude. When the window size grows (cf. Fig. 19b), removing the candidate index leads to poor performance. The gain of the incremental stock maintenance is almost independent of the window size.

Summarizing, the performance of SWOOP is mainly due to (a) the candidate index, which controls the number of pre-candidates as the window size increases, and (b) the incremental stock maintenance, which is up to an order of magnitude faster than recomputing the stock from scratch.

**Static algorithm.** Static does not maintain a stock. Instead, the join result is computed from scratch, including building a new index, whenever the sliding window changes. This approach does not scale to large window sizes since the join time depends on the number of sets in the window.

Note that Static cannot process new sets in batches. Each new set that enters the window may change all values of the top-*k* result. Therefore, an approximate solution that processes batches of size $b>1$ ($b=1$ is the exact algorithm) may introduce a large error. The error rate, measured as the ratio between windows with the correct vs. windows with an incorrect top-*k* results, is $O(1-1/b)$. The error is also high in practice. For example, the error is 65% for batch size $b=100$ on ENRON ($|W|=1000$, $k=10$); more than 75% of the incorrect top-*k* lists differ by more than one element.

### Latency

To study the latency of SWOOP, we modified the timestamps in the TWEET dataset in order to produce a stream with a constant number of sets per second. We load SWOOP with 80% of the average set rate for the respective window size and measure the latency. The latencies are small. For $\overline{|W|}=10^4$ ($4.72\cdot 10^4$ sets/second), the maximum latency is 0.25s with a maximum queue of 12,015 sets. For $\overline{|W|}=10^6$ ($3.97\cdot 10^4$ sets/second), the maximum latency is 0.05s with a maximum queue length of 1714 sets. Interestingly, the latency is lower for larger windows. We attribute this effect to the skyband lower bound, which is looser for small windows (and fewer pairs in the stock). This may lead to more pre-candidates for individual sets. In fact, the maximum processing time (candidate generation plus stock update) of a set is 0.04s for $\overline{|W|}=10^6$ and 0.10s for $\overline{|W|}=10^4$. This effect is limited to individual sets and does not show in the overall number of pre-candidates (cf. Fig. 18a). Table 7 reports the latencies of SWOOP and its competitors for varying set rates and window sizes $\overline{|W|}$. For $\overline{|W|}=10^6$, Static and SCase run into a timeout and are omitted in the table.

**Table 7:** Maximum latency and queue length (TWEET with a constant number of sets per second)

Algorithm	Max. latency	Max. queue length
*80% of the avg. set rate of SWOOP*, $\overline{|W|}=10^4$		
SWOOP	0.25s	12,015
Static	6321 s	28,726,155
SCase	92,435 s	34,174,022
*5% of the avg. set rate of SWOOP*, $\overline{|W|}=10^4$		
SWOOP	0.25s	12,015
Static	29.61s	2323
SCase	78,784 s	28,883,520
*80% of the avg. set rate of SWOOP*, $\overline{|W|}=10^6$		
SWOOP	0.05s	1,714
*5% of the avg. set rate of SWOOP*, $\overline{|W|}=10^6$		
SWOOP	0.03s	68

**Fig. 20:**
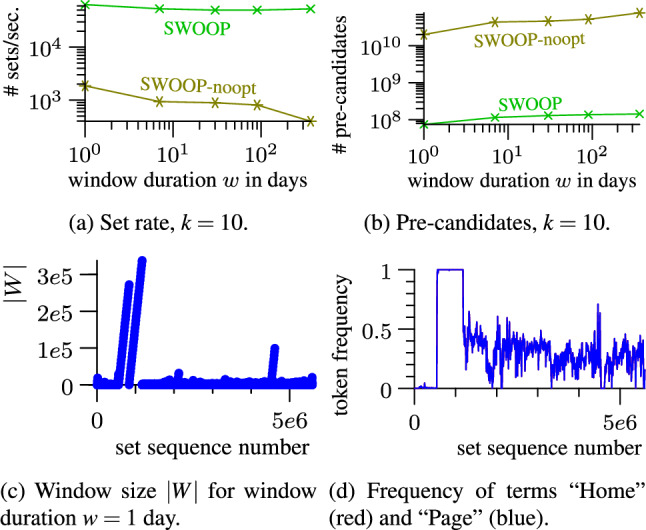
Optimized token processing order (DBLP)

### Optimized token processing order

We measure the effect of the processing order of the index lists during candidate generation in SWOOP. This is relevant only to SWOOP since SCase and Base do not use an index, and the processing order in Static cannot be changed.

In Sect. 6.5, we propose to process the index lists in ascending order of their length. We compare SWOOP, which uses this optimization, to SWOOP-noopt that uses the token order established based on the first appearance of a token on the stream.

We execute the experiment on all datasets. For TWEET, FLICKR, and ENRON, we see almost no runtime difference, indicating that the token order is a good estimate of the real frequency in the stream. The picture is different for DBLP. Figure 20a shows that SWOOP can process the DBLP stream at a rate between 36 and 83 times faster than SWOOP-noopt. The reason is the skew in the DBLP dataset.

First, the sets are received in the stream at a very irregular rate, such that the window size |*W*| varies between 0 and 338,199 for $w=1$ day (cf. Fig. 20c). For large window sizes, the index lists grow long, and a poor list order has major effects on the performance.

Second, the tokens ’Page’ and ’Home’ are only introduced at the time points 2018 and 9764, respectively. However, these tokens become very frequent later (between 10 and 50% for most of the stream), as Fig. 20d shows (due to high correlation, the blue curve for ’Page’ almost exactly tracks the red curve of ’Home’). As a result, these tokens get assigned token numbers for infrequent tokens. Even worse, the largest frequency (almost 100%) of these tokens occurs during the spikes in the window size, leading to very large numbers of pre-candidates (cf. Fig. 20b).

This offers empirical evidence that the optimization of the token order is relevant for difficult streams that are highly skewed.

**Fig. 21:**
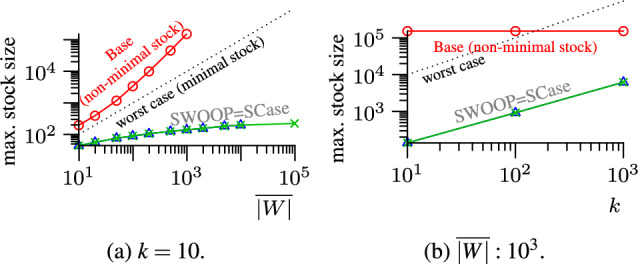
Maximum stock size in *k* and $\overline{|W|}$ (FLICKR)

### Stock size

We study the maximum stock size for SWOOP, SCase, and Base. Specifically, we consider the maximum number of pairs that were stored in the stock during the processing of a particular stream. The stock size of Base is quadratic in the window size $\overline{|W|}$, as it stores all pairs (with non-zero overlap) in the window. The stocks of both SWOOP and SCase are minimal and of size $O(k\cdot |W|)$ in the worst case. Figure 21 shows the stock size for increasing window sizes $\overline{|W|}$ and increasing values of *k*. As expected, the stock size of Base grows fast with the window size. Interestingly, the stock size of SWOOP and SCase grows much slower than the worst case of a minimal stock (indicated by the dotted lines). The stock size of Base is independent of *k*, as it stores all pairs (with non-zero overlap)—see Fig. 21b. The minimal stock of SWOOP and SCase is well below the worst case and also grows slowly. At $k=10$, the maximum stock size is $1.5\cdot 10^2$, while at $k=1000$, it is $6\cdot 10^3$, which is substantially below the worst case minimal stock size.

These results are in line with the previous findings [37], where the asymptotic behavior of the *expected* stock size is shown to be $O(k\cdot \log (\overline{|W|}/k))$. Overall, the advantage of maintaining a minimal stock is clearly supported by our experiments.

### Memory consumption

**Fig. 22:**
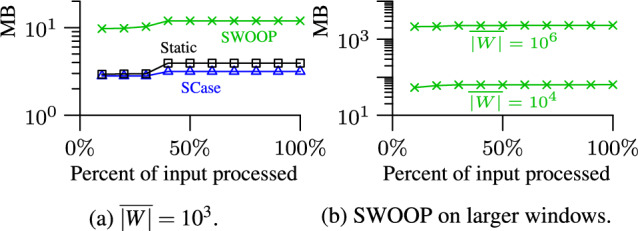
Peak heap memory, TWEET, $k=100$

We study the memory consumption of Static, SCase, and SWOOP over time and measure the peak heap memory after processing *x* percent of the TWEET dataset, $x\in \{10,20,\ldots ,100\}$. For SWOOP, the measurement includes the candidate index, the stock, and the end time index. The stock and the end time index have the same size and grow linearly in both the window size and *k* (cf. Sect. 7.1); the candidate index depends only on the aggregated set sizes in the window and is independent of *k* (cf. Sect. 6.1). As we use a time-based window, such that the number of sets in the window varies over time, we expect also the peak memory consumption to vary. However, the memory consumption should not grow with the stream length. As Fig. 22 shows, the memory consumption is stable for all tested algorithms and does not grow with the stream length. For SWOOP and a window size $\overline{|W|}=10^4$, the difference between the largest and smallest value is $23\%$; for $\overline{|W|}=10^6$, the difference is at most $7\%$. We conclude that SWOOP has a stable memory consumption and does not accumulate memory over time.

## Conclusion and future work

We presented SWOOP, a novel algorithm for continuous top-*k* similarity joins over streams of sets. We introduced the notion of well-behaved similarity function to characterize the class of supported similarity functions. SWOOP integrates new set-based optimizations and a novel, incremental technique to maintain the join result. An extensive empirical comparison offers evidence that SWOOP outperforms the state-of-the-art algorithm SCase and a baseline by up to three orders of magnitude (on inputs for which the competitors do not time out).

SWOOP is a sequential algorithm with two building blocks: the candidate index and the stock. Interesting future work extends SWOOP to parallel or distributed settings. Updating and querying the candidate index in parallel for a new event on the stream requires only minor changes to the algorithm. The candidate index is implemented using inverted lists, and insertions and deletions on different lists are independent. Querying the lists involves computing the similarities for all candidate pairs, which lends itself to parallelization; since different lists may produce identical candidate pairs, the candidates from different compute threads must be deduplicated. A distributed implementation of the candidate index requires updatable, distributed inverted lists as have recently been proposed by Widmoser et al. [44]. Parallelizing or distributing SWOOP’s stock update is more challenging because our merge-insert algorithm relies on the order in which the candidates are inserted.

## Footnotes

1 Note that in rapid streams the number of sets in a window may be large also for short windows.

2 http://www.daisy.aau.dk/.

3 http://dblp.uni-trier.de/.

4 Provided by Bouros et al. [5].

5 http://www.boost.org/.

6 Because Base does not use an index, the pre-candidates include set pairs that do not share any tokens. The candidates of Base are set pairs with similarity greater than zero.

## References

[1] SWOOP: Source code and datasets (2024). https://www.wm1.at/paper/swoop/

[2] Amagata, D., Hara, T., Xiao, C.: Dynamic set kNN self-join. In: Proc. ICDE, pp. 818–829 (2019). 10.1109/ICDE.2019.00078

[3] Bayardo, R.J., Ma, Y., Srikant, R.: Scaling up all pairs similarity search. In: Proceedings of the 16th International Conference on World Wide Web, pp. 131–140 (2007). 10.1145/1242572.1242591

[4] Böhm, C., Ooi, B.C., Plant, C., Yan, Y.: Efficiently processing continuous k-NN queries on data streams. In: Proc. ICDE, pp. 156–165 (2007). 10.1109/ICDE.2007.367861

[5] Bouros, P., Ge, S., Mamoulis, N.: Spatio-textual similarity joins. PVLDB **6**(1), 1–12 (2012). 10.14778/2428536.2428537

[6] Burdisso, S.G., Errecalde, M., y Gómez, M.M.: -SS3: a text classifier with dynamic n-grams for early risk detection over text streams. Pattern Recogn. Lett. **138**, 130–137 (2020). 10.1016/j.patrec.2020.07.001

[7] Chaudhuri, S., Ganti, V., Kaushik, R.: A primitive operator for similarity joins in data cleaning. In: 22nd International Conference on Data Engineering, pp. 5–16 (2006). 10.1109/ICDE.2006.9

[8] Cheema, M.A., Lin, X., Wang, H., Wang, J., Zhang, W.: A unified approach for computing top-k pairs in multidimensional space. In: IEEE 27th International Conference on Data Engineering, pp. 1031–1042 (2011). 10.1109/ICDE.2011.5767903

[9] Cheema, M.A., Lin, X., Wang, H., Wang, J., Zhang, W.: A unified framework for answering k closest pairs queries and variants. IEEE TKDE **26**(11), 2610–2624 (2014). 10.1109/TKDE.2014.2304469

[10] De Francisci Morales, G., Gionis, A.: Streaming similarity self-join. PVLDB **9**(10), 792–803 (2016). 10.14778/2977797.2977805

[11] Deng, D., Li, G., Wen, H., Feng, J.: An efficient partition based method for exact set similarity joins. PVLDB **9**(4), 360–371 (2015). 10.14778/2856318.2856330

[12] Dewang, R.K., Singh, A.K.: State-of-art approaches for review spammer detection: a survey. J. Intell. Inf. Syst. **50**, 231–264 (2018). 10.1007/s10844-017-0454-7

[13] Fedoryszak, M., Frederick, B., Rajaram, V., Zhong, C.: Real-time event detection on social data streams. In: Proceedings of the 25th ACM SIGKDD International Conference on Knowledge Discovery & Data Mining, pp. 2774–2782 (2019). 10.1145/3292500.3330689

[14] Hariharan, B., Jamal, N., Kundu, A., Ramarao, V.T., Risher, M.E., Xi, X., Zheng, L.: Detecting bulk fraudulent registration of email accounts (2014). US Patent 8826450. https://patents.google.com/patent/US20100076922A1/en

[15] Hong, M., Demers, A., Gehrke, J., Riedewald, M.: Event and Pattern Detection over Streams, pp. 1354–1358. Springer (2018). 10.1007/978-1-4614-8265-9_155

[16] Hu, H., Li, G., Bao, Z., Feng, J., Wu, Y., Gong, Z., Xu, Y.: Top-k spatio-textual similarity join. IEEE TKDE **28**(2), 551–565 (2016). 10.1109/TKDE.2015.2485213

[17] Ilyas, I.F., Aref, W.G., Elmagarmid, A.K.: Supporting top-k join queries in relational databases. VLDB J. **13**(3), 207–221 (2004). 10.1007/s00778-004-0128-2

[18] Jiang, H., Zhu, R., Wang, B.: EPF: a general framework for supporting continuous top-k queries over streaming data. Cognit. Comput. **12**, 176–194 (2020). 10.1007/s12559-019-09661-z

[19] Jung, A., Mirbabaie, M., Ross, B., Stieglitz, S., Neuberger, C., Kapidzic, S.: Information diffusion between twitter and online media. In: Proceedings of the International Conference on Information Systems - Bridging the Internet of People, Data, and Things (2018). https://aisel.aisnet.org/icis2018/bridging/Presentations/6

[20] Klimt, B., Yang, Y.: Introducing the enron corpus. In: Proceedings of the Conference on Email and Anti-Spam (2004). http://www.ceas.cc/papers-2004/168.pdf

[21] Kocher, D., Augsten, N.: A scalable index for top-k subtree similarity queries. In: Proceedings of the ACM SIGMOD, pp. 1624–1641 (2019). 10.1145/3299869.3319892

[22] Ley, M.: DBLP-some lessons learned. PVLDB **2**(2), 1493–1500 (2009). 10.14778/1687553.1687577

[23] Mann, W., Augsten, N.: PEL: Position-enhanced length filter for set similarity joins. In: Proceedings of the Foundations of Databases, pp. 89–94 (2014). https://ceur-ws.org/Vol-1313/paper_16.pdf

[24] Mann, W., Augsten, N., Bouros, P.: An empirical evaluation of set similarity join techniques. Technical Report University of Salzburg, Austria (2015). http://ssjoin.dbresearch.uni-salzburg.at/

[25] Mann, W., Augsten, N., Bouros, P.: An empirical evaluation of set similarity join techniques. PVLDB **9**(4), 360–371 (2015). 10.14778/2947618.2947620

[26] Mellin, J., Berndtsson, M.: Event Detection, pp. 1361–1366. Springer (2018). 10.1007/978-1-4614-8265-9_506

[27] Mislove, A., Marcon, M., Gummadi, K.P., Druschel, P., Bhattacharjee, B.: Measurement and analysis of online social networks. In: Proceedings of the 7th ACM SIGCOMM Conference on Internet Measurement, pp. 29–42 (2007).10.1145/1298306.1298311

[28] Mouratidis, K., Bakiras, S., Papadias, D.: Continuous monitoring of top-k queries over sliding windows. In: Proceedings of the 7th ACM SIGCOMM Conference on Internet Measurement, pp. 635–646 (2006). 10.1145/1142473.1142544

[29] Pacífico, L., Ribeiro, L.A.: SSTR: Set similarity join over stream data. In: Proceedings of the International Conference on Enterprise Information Systems, pp. 52–60 (2020). 10.5220/0009420400520060

[30] Pacífico, L., Ribeiro, L.A.: Streaming set similarity joins. In: Enterprise Information Systems, pp. 24–42 (2021). 10.1007/978-3-030-75418-1_2

[31] Papadias, D., Tao, Y., Fu, G., Seeger, B.: Progressive skyline computation in database systems. ACM TODS **30**(1), 41–82 (2005). 10.1145/1061318.1061320

[32] Pripužić, K., Žarko, I.P., Aberer, K.: Time- and space-efficient sliding window top-k query processing. ACM TODS **40**(1), 1:1-1:44 (2015). 10.1145/2736701

[33] Qi, S., Bouros, P., Mamoulis, N.: Top-k string similarity joins. In: Proceedings of the 32nd International Conference on Scientific and Statistical Database Management (2020). 10.1145/3400903.3400922

[34] Requena, B., Cassani, G., Tagliabue, J., Greco, C., Lacasa, L.: Shopper intent prediction from clickstream e-commerce data with minimal browsing information. Sci. Rep. **10**, 16983 (2020). 10.1038/s41598-020-73622-y 33046722 10.1038/s41598-020-73622-y PMC7550603

[35] Ribeiro, L.A., Härder, T.: Generalizing prefix filtering to improve set similarity joins. Inf. Syst. **36**(1), 62–78 (2011). 10.1016/j.is.2010.07.003

[36] Shen, Z., Cheema, M.A., Lin, X., Zhang, W., Wang, H.: Efficiently monitoring top-k pairs over sliding windows. In: 2012 IEEE 28th International Conference on Data Engineering , pp. 798–809. IEEE (2012). 10.1109/ICDE.2012.89

[37] Shen, Z., Cheema, M.A., Lin, X., Zhang, W., Wang, H.: A generic framework for top-k pairs and top-k objects queries over sliding windows. IEEE TKDE **26**(6), 1349–1366 (2014). 10.1109/TKDE.2012.181

[38] Wahab, R.A.S.R., Rum, S.N.M., Ibrahim, H., Sidi, F., Ishak, I.: A method for processing top-k continuous query on uncertain data stream in sliding window model. WSEAS Trans. Syst. Control **16**, 22 (2021). 10.37394/23203.2021.16.22

[39] Wang, G., Zhang, X., Tang, S., Wilson, C., Zheng, H., Zhao, B.Y.: Clickstream user behavior models. ACM Trans. Web **11**(4), 1–37 (2017). 10.1145/3068332

[40] Wang, H., Yang, L., Xiao, Y.: SETJoin: a novel top-k similarity join algorithm. Soft Comput. (2020). 10.1007/s00500-020-04807-w

[41] Wang, J., Li, G., Feng, J.: Can we beat the prefix filtering?: An adaptive framework for similarity join and search. In: Proceedings of the 2012 ACM SIGMOD International Conference on Management of Data, pp. 85–96 (2012). 10.1145/2213836.2213847

[42] Wang, P., Xiao, C., Qin, J., Wang, W., Zhang, X., Ishikawa, Y.: Local similarity search for unstructured text. In: Proceedings of the 2016 International Conference on Management of Data, pp. 1991–2005 (2016). 10.1145/2882903.2915211

[43] Wang, X., Qin, L., Lin, X., Zhang, Y., Chang, L.: Leveraging set relations in exact set similarity join. PVLDB **10**(9), 925–936 (2017). 10.14778/3099622.3099624

[44] Widmoser, M., Kocher, D., Augsten, N.: Scalable distributed inverted list indexes in disaggregated memory. Proc. ACM Manag. Data (2024). 10.1145/3654974

[45] Xiao, C., Wang, W., Lin, X., Shang, H.: Top-k set similarity joins. In: 2009 IEEE 25th International Conference on Data Engineering, pp. 916–927 (2009). 10.1109/ICDE.2009.111

[46] Xiao, C., Wang, W., Lin, X., Yu, J.X.: Efficient similarity joins for near duplicate detection. In: Proceedings of the 17th International Conference on World Wide Web, pp. 131–140 (2008). 10.1145/1367497.1367516

[47] Xiao, C., Wang, W., Lin, X., Yu, J.X., Wang, G.: Efficient similarity joins for near-duplicate detection. ACM TODS **36**(3), 1–41 (2011). 10.1145/2000824.2000825

[48] Xiao, C., Wang, W., Lin, X., Yu, J.X., Wang, G.: Efficient similarity joins for near-duplicate detection. ACM TODS **36**(3), 1–41 (2011). 10.1145/2000824.2000825

[49] Xu, X., Gao, C., Pei, J., Wang, K., Al-Barakati, A.: Continuous similarity search for evolving queries. Knowl. Inf. Syst. **48**(3), 649–678 (2016). 10.1007/s10115-015-0892-x

[50] Yamazaki, T., Koga, H.: Exact algorithm to solve continuous similarity search for evolving queries and its variant. IEICE Trans. Inf. Syst. **E105.D**(5), 898–908 (2022). 10.1587/transinf.2021DAP0003

[51] Yang, D., Shastri, A., Rundensteiner, E.A., Ward, M.O.: An optimal strategy for monitoring top-k queries in streaming windows. In: Proceedings of the 14th International Conference on Extending Database Technology, pp. 57–68 (2011). 10.1145/1951365.1951375

[52] Yang, J., Zhang, W., Wang, X., Zhang, Y., Lin, X.: Distributed streaming set similarity join. In: 2020 IEEE 36th International Conference on Data Engineering (ICDE), pp. 565–576 (2020). 10.1109/ICDE48307.2020.00055

[53] Yang, Z., Zheng, B., Li, G., Zhao, X., Zhou, X., Jensen, C.S.: Adaptive top-k overlap set similarity joins. In: 2020 IEEE 36th International Conference on Data Engineering (ICDE), pp. 1081–1092 (2020). 10.1109/ICDE48307.2020.00098

[54] Zhang, W., Xu, J., Liang, X., Zhang, Y., Lin, X.: Top-k similarity join over multi-valued objects. In: International Conference on Database Systems for Advanced Applications, pp. 509–525 (2012). 10.1007/978-3-642-29038-1_37

[55] Zheng, Z., Kohavi, R., Mason, L.: Real world performance of association rule algorithms. In: Proceedings of the Seventh ACM SIGKDD International Conference on Knowledge Discovery and Data Mining, pp. 401–406 (2001).10.1145/502512.502572

[56] Zhu, E., Deng, D., Nargesian, F., Miller, R.J.: JOSIE: Overlap set similarity search for finding joinable tables in data lakes. In: Proceedings of the 2019 International Conference on Management of Data, pp. 847–864 (2019). 10.1145/3299869.3300065

[57] Zhu, M., Lee, D.L., Zhang, J.: k-closest pair query monitoring over moving objects. In: Proceedings of the International Conference on Mobile Data Management, pp. 14–14 (2006). 10.1109/MDM.2006.99

[58] Zhu, R., Meng, L., Wang, B., Yang, X., Xia, X.: Approximate continuous top-k queries over memory limitation-based streaming data. In: International Conference on Database Systems for Advanced Applications, pp. 3–20 (2022). 10.1007/978-3-031-00123-9_1

[59] Zhu, R., Wang, B., Yang, X., Zheng, B., Wang, G.: SAP: improving continuous top-k queries over streaming data. IEEE TKDE **29**(6), 1310–1328 (2017). 10.1109/TKDE.2017.2662236

[60] Zois, V., Tsotras, V.J., Najjar, W.A.: Efficient main-memory top-k selection for multicore architectures. PVLDB **13**(2), 114–127 (2019). 10.14778/3364324.3364327

